# Guidelines and Preliminary Results of Group-Based Nutrition Interventions for Obesity Management Among Adults in Brazilian Primary Health Care

**DOI:** 10.3390/ijerph22071093

**Published:** 2025-07-09

**Authors:** Angélica Ribeiro e Silva, Maria Cecília Ramos de Carvalho, Nathália Luíza Ferreira, Natacha Toral, Camila Kümmel Duarte, Aline Cristine Souza Lopes

**Affiliations:** 1 Grupo de Pesquisa de Intervenções em Nutrição (GIN/UFMG), Nursing School, Universidade Federal de Minas Gerais, Belo Horizonte 30130-100, MG, Brazil; angelicarsnutri@gmail.com (A.R.e.S.); ceciliaramosnutri@gmail.com (M.C.R.d.C.); camila.kummel@gmail.com (C.K.D.); 2 Núcleo de Estudos e Pesquisas em Epidemiologia e Nutrição (NEPEN), Grupo de Pesquisa de Intervenções em Nutrição (GIN/UFMG), Universidade Federal de Lavras, Lavras 37200-000, MG, Brazil; nathalia.luiza@ufla.br; 3 Núcleo de Estudos Epidemiológicos em Saúde e Nutrição (NESNUT), Grupo de Pesquisa de Intervenções em Nutrição (GIN/UFMG), Campus Universitário Darcy Ribeiro, Universidade de Brasília, Brasília 70910-900, DF, Brazil; natachatoral@unb.br

**Keywords:** health education, primary health care, obesity management, controlled clinical trial

## Abstract

This study presents the guideline and preliminary results of a randomized controlled community trial of group-based nutrition interventions for obesity management in Brazilian Primary Health Care (PHC), occurring between 2022 and 2023. Belo Horizonte PHC units were randomly sampled and allocated to the control (CG) or intervention group (IG). Participants were those in preparation with high self-efficacy, action, or maintenance stages of change for weight reduction who were willing to participate in groups. CG participants received usual care, while IG participants were allocated into two therapeutic groups (TGs): non-severe (TG1) or severe obesity (TG2). We analyzed demographics data, stages of change and self-efficacy for weight reduction, and intervention adherence. The six-month theory-based interventions included face-to-face groups (TG1:7; TG2:9) and phone messages and postcards delivered (TG1:4; TG2:5). There were 1120 participants. Most were women, with a median age of 61.4 years; 45.4% were in the maintenance stage of change for weight reduction, and 76.1% had high self-efficacy. A total of 350 groups were held, 13,473 phone calls made, and 1973 postcards delivered. The median adherence was 52.4%. The reach and adherence to groups demonstrate their viability and scalable potential for the treatment of obesity in Brazilian PHC, contributing to the strengthening of chronic disease management actions.

## 1. Introduction

Obesity affects 800 million people worldwide [1], with a prevalence of 10.8% among men and 14.9% among women [2]. From 2006 to 2023, in Brazil, the prevalence of obesity in adults living in capitals increased from 11.8% to 24.3% [3]. So, effective lifestyle interventions are urgent to address obesity [4,5]. Primary Health Care (PHC) plays a fundamental role in this context due to its attributes of first contact, care coordination, comprehensiveness, and continuity of care [6]. Furthermore, it is a privileged setting for health education and intersectoral articulation, which are essential for obesity management [5,7].

PHC allows the delivery of tailored obesity management strategies according to an individual’s experiences and socioeconomic status, severity of obesity [8], and comorbidities, and their readiness and self-efficacy to change health behaviors [7,9]. A systematic review with meta-analysis revealed an average weight reduction of 3.2 kg (±6.4) among overweight individuals after 12 months of a PHC-based intervention, compared to 1.2 kg (±6.0) in the control group. A significant reduction in waist circumference was also observed at 12 and at 24 months, and the intervention group maintained a mean difference of −1.8 kg compared to the control group [10]. Maintaining weight loss and behavior changes over the long term is a major challenge in managing obesity. While many interventions successfully lead to initial weight loss, individuals often struggle to sustain these results, resulting in weight regain. However, a study showed that participants in a group intervention within PHC were able to maintain their weight loss compared to the control group even after 24 months. This finding highlights the significant potential of such interventions to promote lasting results.

Despite the potential of PHC interventions for obesity management, PHC professionals face challenges to deliver effective obesity care. These challenges include insufficient professional qualification, the complexity of care when comorbidities are present, a high demand for individual assistance, and a scarcity of instructional materials [11].

The Brazilian Ministry of Health has published instructional manuals for obesity management within the Unified Health System (SUS—*Sistema Único de Saúde*); however, there is a gap between public policy formulation and in the service implementation due to the complexities of Brazilian PHC. Multi-component group-based interventions can help overcome these challenges as they assist a large number of individuals, optimize human and material resources, and provide opportunities for professionals and individuals to build rapport and share experiences [7,12]. These interventions have a higher rate of achieving ≥5% body weight reduction after 12 months compared to individual interventions [12]. Furthermore, Brazilian PHC is a potential setting in which to expand group-based obesity management, since less than 60% of PHC centers routinely deliver this type of intervention [13].

However, high-quality reports of group-based nutrition interventions for obesity management in PHC are scarce [14]. The interventions usually lack detail and scientific rigor, hindering their reproducibility and use [15,16]. Intervention duration and sample size are also suboptimal, with most studies presenting a sample size of fewer than 200 individuals [17,18]. Most of the studies have reported a follow-up of 12 weeks or less [17], although interventions that last for 6 to 12 months are more effective for sustainable weight reduction [16].

Furthermore, the use and assessment of information and communication technologies (ICTs) in obesity management have also been incipient despite their potential to increase adherence to lifestyle interventions [9,18,19] and provide access to information and communication across geographical boundaries. ICTs refer to a variety of tools and systems that facilitate communication and the exchange of information. This includes both hardware and software, such as radio, television, mobile phones, computers, network infrastructure, and satellite systems, and services like videoconferencing and distance learning platforms [20].

The present study used ICTs and face-to-face nutrition interventions, based on theories for health behavior change and health education. The theories utilized were the Transtheoretical Model, Cognitive Behavioral Therapy (CBT), and the critical-reflective approach proposed by Paulo Freire. This choice is based on studies that demonstrate that the use of ICTs and the adoption of theories can increase the effectiveness of interventions related to obesity management [9].

The Transtheoretical Model assumes that health behavior change occurs in steps. This theory is based on the following four pillars: stages of change (pre-contemplation, contemplation, preparation/decision, action, and maintenance), which refer to an individual’s readiness to change their behavior; decisional balance, which is the individual perception of the advantages and disadvantages of behavior change; self-efficacy, which means the extent to which the person feels capable of changing and maintaining a new behavior; and processes of change (cognitive and behavioral), which are individual actions that assist in the progression through the stages of change [21]. Already, according to CBT, individuals develop and maintain beliefs from which they formulate their views of themselves, the world, and the future [22]. Thus, the CBT seeks to modify unhealthy beliefs and cognitive distortions and to develop skills to reduce the risk of relapse [23]. And the critical-reflective approach facilitates health behavior changes by being based on the construction of protagonism, empowerment, and autonomy of people [24].

Thus, this study aimed to present the guidelines and activities carried out in a randomized controlled community trial (RCCT) encompassing two group-based nutrition interventions for obesity management, according to the severity of obesity, among individuals in Brazilian PHC services. Furthermore, we present preliminary results on baseline characteristics of the sample, differences between control and intervention group participants, and nutrition intervention adherence rate. We hope that the collective intervention approach for managing obesity, based on the theories mentioned, will be effectively embraced by users and that PHC professionals will implement it among the strategies aimed at controlling body weight.

## 2. Methods

This study was carried out with a representative sample of the Health Academy Program (PAS—*Programa Academia da Saúde*) in Belo Horizonte, Minas Gerais, Brazil. Participants with obesity from PAS units randomized to the control group (CG) only received usual care, which consists of supervised collective physical exercise for one hour, three times a week, and health education on different topics [25], not including food and nutrition education aimed at obesity management. In addition to usual care, participants in the intervention group (IG) participated in one of two group-based nutrition interventions called therapeutic groups (TGs), based on behavior change theories [26] and tailored to the severity of obesity.

### 2.1. Study Setting and Participants

Belo Horizonte has an estimated population of 2,315,560 inhabitants [27], a high Human Development Index of 0.797 [28], and is divided into nine administrative districts [29].

PAS was implemented in Belo Horizonte in 2006 [30], is part of the Brazilian Unified Health System, and inspired a national program implemented by the Brazilian Ministry of Health in 2011. PAS units are public facilities within the PHC network, where regular exercise and health education are offered free of charge to the population, focusing on health promotion, disease prevention, and control of non-communicable diseases [31].

### 2.2. Sample Size

The sampling process was carried out by a statistician specifically hired for this purpose, ensuring that the researchers were not involved in selecting the PAS units or in assigning participants to the IG and CG.

Among the 78 existing PAS units in Belo Horizonte during the sampling period (2019), those that did not offer group-based obesity management in the previous two years and operated in the morning were deemed eligible for sampling. There were not enough units operating in other shifts to allow pairing in the regions, which could create an imbalance in the sample due to the varied characteristics of users participating in different shifts.

A simple random sampling method was employed, stratified by the nine administrative districts of the city. The following parameters were used for sample size estimation: a 5% difference in weight loss between groups, 80% sampling power, a 35% increase in sample size to account for potential losses to follow-up, and a 20% prevalence of obesity.

A two-stage random number table was utilized during the sampling process. In the first stage, two units were selected from each administrative district. In the second stage, units were randomly assigned to either the GC or the IG within the RCCT design. In this design, each PAS unit represents a cluster of participants, and a specific number of units were chosen in each stratum (administrative district) until the desired sample size was reached. The final sample consisted of 1263 program users. In each stratum (administrative district), the number of PAS units needed to complete the designated sample size was selected, corresponding to 59 PAS units. These units were then allocated to form the study groups (CG or IG) using Simple Stratified Random Sampling (Supplementary Material Table S1). The total of PAS units of this study achieved a final sampling power of 97.2%.

In each sampled PAS unit, we interviewed individuals aged 20 years or older who had attended at least one physical exercise class in the previous month. Pregnant women and individuals with cognitive impairments, determined by their difficulty in understanding and answering questions as well as medical diagnoses, were excluded from participating in data collection.

### 2.3. Participant Recruitment and Data Collection

Participant recruitment and data collection were performed in four stages: screening, baseline, nutrition interventions, and reassessment. Occurring between 2022 and 2023.

Before data collection began at the PAS units, the research was presented to users along with an explanation of the importance of their collective participation. Following this presentation, individuals were invited to participate based on established inclusion and exclusion criteria.

Data collection took place during the operating hours of the PAS units. Teams consisted of health professionals and undergraduate students, who were assigned roles as field supervisors and interviewers (typically five on average), along with a general field supervisor and research coordinators. The interviewers were responsible for addressing any doubts, obtaining informed consent, and conducting face-to-face individual interviews throughout the research process. Field supervisors were tasked with initiating data collection by contacting the PAS units, ensuring the consistency of questionnaires, performing anthropometric assessments of participants, scheduling and coordinating interviews, recording absences, refusals, and exclusions, and submitting weekly field reports.

All team members received training, and equipment was calibrated prior to the start of data collection. Manuals detailing data collection protocols and field logistics were provided to assist teams in the field. Training and equipment calibration will occur every six months or whenever specific needs arise.

Electronic tablets were used for the interviews, with questionnaires available in the Epicollet5 Data Collection application (Oxford University Big Data Institute, CGPS team, Oxford, UK). If the tablets were unstable, the information was collected on paper and typed by trained typists using the same application. The data were collected in a private location, and each participant received an identification number to ensure anonymity. Furthermore, the tablets were accessed through passwords. More details can be found in the protocol published by Freitas and collaborators [32].

For the screening stage we adapted the “Group Stratification for Obesity Management”, proposed in the “Instructive of Collective Approach for the obesity management in SUS” [7], aimed at identifying individuals eligible for obesity treatment. This Instructive was developed by the Research Group on Nutrition Interventions at the Federal University of Minas Gerais, in partnership with the Brazilian Ministry of Health. Its aim is to support health professionals in delivering group-based interventions for obesity management within the Brazilian Unified Health System. The “Group Stratification for Obesity Management” proposes different types of group interventions based on individuals’ stage of behavior change and self-efficacy for weight reduction, their desire and availability to participate in group-based interventions, and the severity of obesity [7].

We collected data on demographics (sex, age, family income, education, marital status, and professional occupation); anthropometric measurements (weight, height, and waist circumference); self-reported morbidities (high blood pressure, dyslipidemia, joint disease, diabetes, cardiovascular disease, and sleep apnea) [33]; constructs of the Transtheoretical Model (stages of behavior change and self-efficacy for weight reduction) [7]; and individuals were asked about their desire and availability to participate in a group-based intervention for obesity management for 6 months or longer (Supplementary Material Figure S1).

Weight and height were used to calculate BMI and identify participants with obesity (BMI ≥ 30.0 kg/m^2^) [34]. Calibrated equipment that has been approved through technical certification was utilized for anthropometric assessments. Body weight (in kilograms) was measured using a digital scale with a maximum capacity of 200 kg. Height (in centimeters) was assessed using a portable stadiometer that can measure from 0.35 m to 2.13 m.

An algorithm, proposed by the Brazilian Ministry of Health, was employed to classify each participant into one of the five stages of change for weight reduction: (1) pre-contemplation: people who do not intend to reduce weight in the following six months; (2) contemplation: people who intend to reduce weight in the following six months but have no intention of starting in the following 30 days; (3) preparation: those who intend to start reducing weight in the following 30 days; (4) action: those who report starting changes aimed at weight reduction within the previous six months; (5) maintenance: those who have engaged in behavior change aimed at weight reduction for longer than six months [35].

Self-efficacy for weight reduction was investigated using a validated scale with three items in which the participants indicated their level of confidence for coping with barriers for weight reduction. Self-efficacy was classified as high if the participant reported being very or completely confident to reduce weight in at least two of the three questions and low if the participant was very confident or completely confident in none or only one of the questions [36].

**Figure 1:**
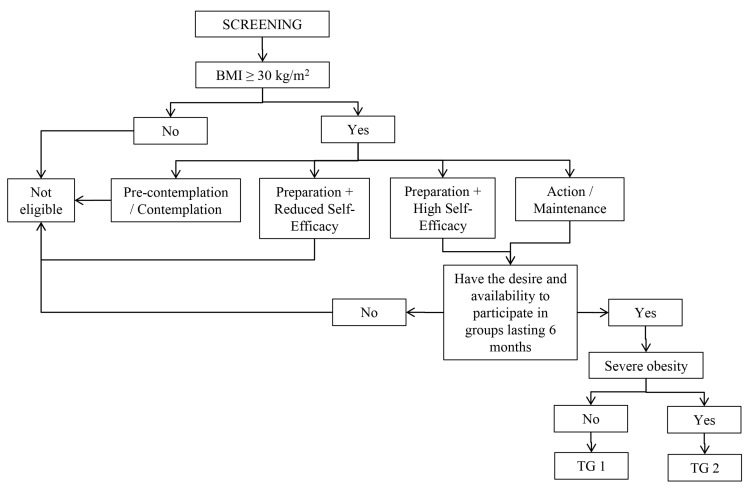
Steps for participant eligibility. Note: BMI = Body Mass Index. TG = Therapeutic Group.

Figure 1 presents the eligibility criteria for participation in TGs. According to the “Group Stratification”, participants who met the following criteria were eligible for baseline data collection: being diagnosed with obesity; being in the preparation stage for weight reduction with high self-efficacy or in the action or maintenance stages regardless of self-efficacy; and having the desire and availability to participate in the group-based intervention [7]. Eligible participants were allocated to one of two TGs according to the severity of obesity, with criteria adapted from national guidelines for surgical obesity treatment and considering comorbidities as markers of severe obesity [33], as recommended by “Strategy for Caring for People with Obesity” proposed in the “Instructive of Collective Approach for the obesity management in SUS” [7]. Individuals with zero or one reported non-communicable disease, except for diabetes mellitus, were considered not to have severe obesity and were allocated to Therapeutic Group 1 (TG1). Those who reported a diagnosis of diabetes mellitus or two or more non-communicable diseases were considered to have severe obesity and allocated to Therapeutic Group 2 (TG2).

Eligible participants participated in a baseline assessment using a questionnaire based on national and international surveys and guidelines from the Brazilian Ministry of Health and the World Health Organization [7,32,37,38]. Baseline data was collected immediately after screening or scheduling. Data on health literacy, food intake, eating practices, motivations for food choices, cooking skills, physical exercise, weight history, weight self-monitoring, and obesity stigma were investigated, as described by Freitas et al. [32] (Supplementary Material Figure S2).

After the nutritional interventions, CG and IG participants from both TG1 and TG2 were reassessed using instruments similar to the screening and baseline stages, with additional data collection on their satisfaction with nutrition interventions in the IG (Supplementary Material Figure S3).

Data underwent consistency analysis immediately after collection and after tabulation by a trained professional. Descriptive analyses were performed to identify any unusual values or responses that fell outside the expected range, as well as to flag any missing data. A document was created detailing all the analyses performed and outlining the decisions made in constructing the final database. The participants’ information was anonymized and only available for research group members under request.

### 2.4. Development of Group-Based Nutrition Interventions

#### 2.4.1. Theorical Intervention Approach

The nutrition interventions offered by the TG1 and TG2 were adapted from the “Instructive of Collective Approach for the obesity management in SUS” [7] and its “Notebook of Educational Activities” [39]. Both TGs were based on theories for health behavior change and health education: the Transtheoretical Model, CBT, and the critical-reflective approach proposed by Paulo Freire.

The four pillars of the Transtheoretical Model were used in our study to plan, develop, and evaluate the intervention and were supported by the critical-reflective approach proposed by Paulo Freire with the objective of building rapport, promoting leadership, increasing empowerment, and facilitating autonomy of people to care for their health [21,40]. In addition, CBT techniques, such as self-monitoring, problem-solving, cognitive restructuring, and social support, were embedded in the scripts for group meetings.

The TGs were developed according to the “Food and Nutrition Education Reference Framework for Public Policies” [40] and the “Dietary Guidelines for the Brazilian Population” [41]. The “Food and Nutrition Education Reference Framework” supports the development, delivery, and assessment of food and nutrition education in Brazilian public services, prioritizing reflective and autonomy-generating interventions [40]. And the Dietary Guidelines define healthy eating based on the degree and extension of industrial food processing [41]. Furthermore, interventions were based on results from a systematic review of clinical trials for obesity management in PHC and secondary health care. In this systematic review, the most effective interventions included continuous contact with participants, employed ICT, had more frequent meetings in the beginning and a reduced frequency over time, and employed behavior change theories and techniques along with guidance on dietary modification delivered by registered dietitians and psychologists [9]. Those characteristics were incorporated in our TGs.

#### 2.4.2. Intervention Team Composition and Training

Nutrition interventions were planned by a multidisciplinary team of dietitians, health educators, psychologists, physical education professionals, and other PHC professionals. The TGs were delivered by three teams made up of dietitians and undergraduate health students (physiotherapy and nursing), supported by a psychologist with experience in groups, PHC, and obesity. Each team was responsible for one group from start to finish.

Before the interventions, team members were trained in teamwork, behavior change theories, and intervention logistics. The training took place over 2 days and was conducted by dietitians with doctorates who were the team supervisors. During the development of the interventions, teams held biweekly meetings to discuss intervention progress, supervised by one psychologist with experience in groups, PHC, and obesity.

#### 2.4.3. Group-Based Nutrition Interventions for Obesity Management

The group-based nutrition interventions for both TG1 and TG2 lasted for six months and included 60-min face-to-face meetings in PAS units and ICT strategies aimed at promoting continued reflection on meeting topics and motivating adherence [7]. The schedule for TG1 included 7 face-to-face meetings and 4 ICT strategies, with the aim of reaching 3% body weight reduction. For TG2, 9 face-to-face meetings and 5 ICT activities were delivered, with the goal of reaching 5% body weight reduction, reflecting a higher level of intervention intensity and the seriousness of the condition of obesity.

Workshops were chosen as the main strategy for face-to-face meetings, aiming to promote knowledge construction through reflection. Each workshop included three sections: initial (participant reception, preparation, and presentation of meeting topics, objectives, and activities), intermediate (diverse interactive strategies to encourage reflection and elaboration on the topics), and systematization (sharing and visualization of the group’s production, with final reflection and synthesis) [42,43]. Environment-based activities, such as collaborative picnics, were also employed to promote critical reflection on behavior change for weight reduction [43].

Postcards and phone calls or messages were the ICT strategies (Supplementary Material Figure S4). Postcards with motivational messages were delivered between meetings to promote participant adherence [43]. Physical education professionals from PAS units were responsible for distributing these cards to users on a date set in advance by the team. Telephone calls and WhatsApp^®^ messages were used by the intervention team to deliver motivational messages, given the potential of these technologies to increase intervention adherence and effectiveness [9]. Detailed themes, objectives, and activities included in TG1 and TG2 are shown in Table 1.

**Figure 2:**
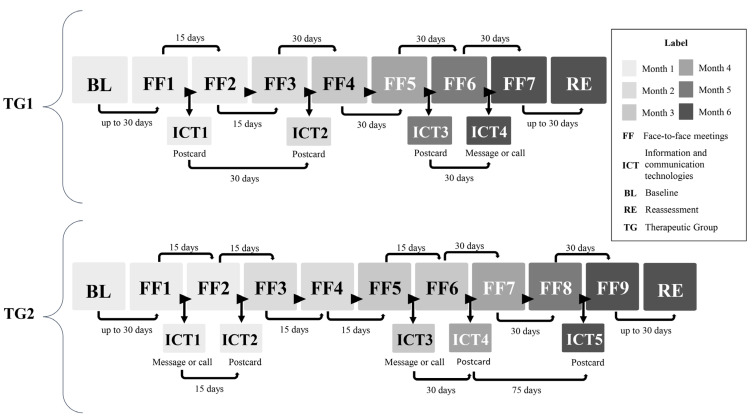
Schedule of face-to-face meetings and ICT strategies throughout the nutrition interventions according to treatment group allocation.

Participants in TG1 and TG2 were advised on cooking skills and portion control, prioritizing the intake of unprocessed and minimally processed foods and reducing the intake of ultra-processed foods, aiming to reduce intake of excess carbohydrates and lipids, and a reduction of 500 to 1000 kcal/day [7]. The intervention schedules for each group are described in Figure 2.

#### 2.4.4. Logistics of Nutrition Interventions

The number of groups to be developed at each PAS unit in the IG was defined according to the number of eligible users for TG1 and TG2. Each group included 8 to 20 participants, and groups were created until all eligible participants were included. If there were less than 8 eligible participants for TG1 in any PAS unit, they were allocated into TG2 and received a more intensive intervention. This adaptation comes from the limitations of holding group meetings with very few participants, which would restrict interactions, social support, and diversity of experiences.

Once screening and baseline data collection at each PAS unit of IG were completed, intervention facilitators contacted PAS teams for a face-to-face meeting. Nutrition interventions began no later than 30 days after the end of baseline data collection. All intervention participants received a reminder via telephone call or message 48 h before each face-to-face meeting. Face-to-face meetings could be rescheduled as long as they respected the limit of seven days before or after the original date.

We employed strategies to anticipate and overcome challenges during intervention delivery. Intervention facilitators met with PAS professionals at each unit before group meetings to identify the optimal day, time, and physical space in which to deliver face-to-face meetings. The PAS professionals were reminded about the importance of delivering postcards to participants.

For the meeting reminders and motivational telephone calls to be successful, up to three attempts to reach each participant were made on different days, including weekends, and if needed, new telephone numbers were sought from PAS professionals.

Adherence data was collected in attendance lists for each group, filled out during each face-to-face meeting. For ICT strategies, spreadsheets were used to control the sending of postcards and the receipt of phone calls or text messages. Total adherence rate to the intervention activities was calculated based on the number of activities each user participated in, divided by the total number of activities offered (11 in TG1 and 14 in TG2). For analysis, adherence was categorized into three levels: up to 30%, between 30% and 70%, and greater than 70% [44].

### 2.5. Ethics Statement

The RCCT received full ethical approval from the Institutional Review Boards of the University (CAAE: 36395320.7.0000.5149; CAAE: 42654421.1.0000.5149) and the City of Belo Horizonte (CAAE: 36395320.7.3001.5140; CAAE: 42654421.1.3001.5140). It was carried out according to the guidelines from the Declaration of Helsinki. All participants were informed by the research team about the objectives and methods of the study, and those who agreed to participate provided consent before participation. The protocols for both RCCTs were registered in the Brazilian Registry of Clinical Trials—(ReBEC) [U1111-1292-3066 (29 May 2021) and U1111-1290-8934 (17 August 2021)].

### 2.6. Statistical Analyses

Data were analyzed on Stata version 14. Data on demographics, self-reported diseases, constructs of the Transtheoretical Model, and adherence rate to the intervention activities were analyzed in this paper. The Shapiro–Wilk test was performed to assess the distribution of continuous data, which was presented as medians and interquartile ranges. Categorical data were presented as percentages with 95% confidence intervals. Mann–Whitney or chi-square tests were performed to identify differences between groups, and *p*-values ≤ 0.05 were considered significant.

## 3. Results

**Figure 3:**
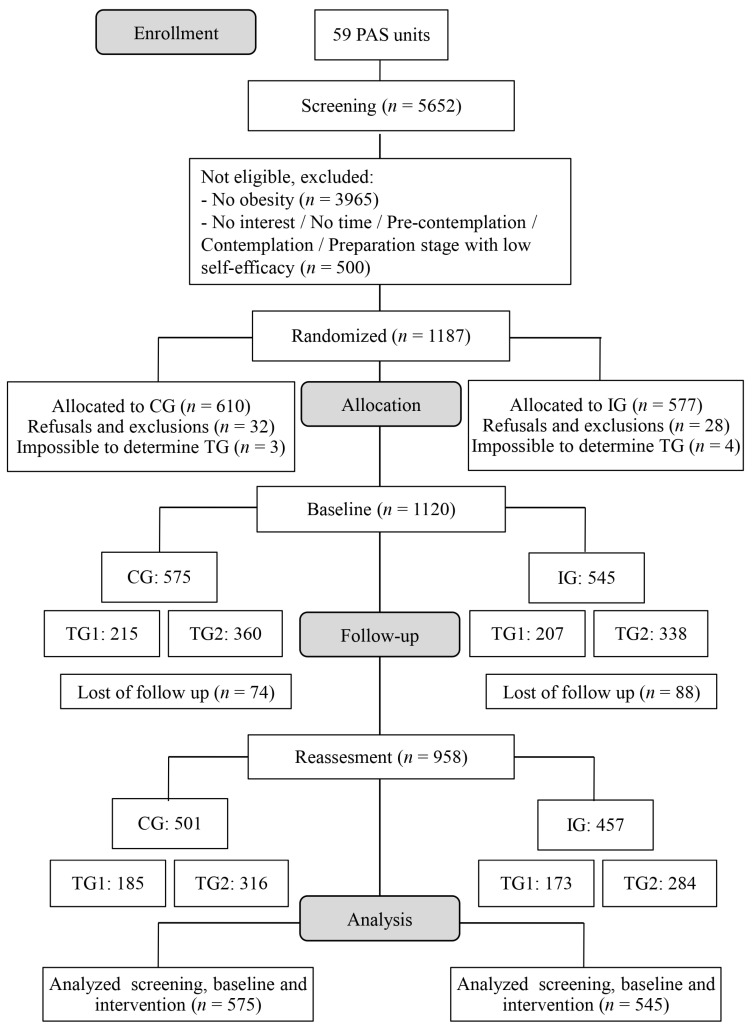
Flowchart of the research steps. Note: PAS = Programa Academia da Saúde; CG = Control Group; IG = Intervention Group; TG = Therapeutic Group.

We evaluated 5652 users in the 59 PAS units. Of these, 3965 were not obese, and 500 were not prepared to treat obesity, according to the Transtheoretical Model, or were interested in participating in groups. So, 1187 were eligible to participate in the group-based nutrition interventions. After refusals and exclusions (*n* = 60) and impossible to determine the TG (*n* = 7), 1120 users were allocated in the groups, according to Figure 1, being 422 in TG1 and 698 in TG2. The reassessment was carried out with 958 participants because of the loss of follow-up (CG = 74; IG = 88), so a total of 358 from TG1 and 600 from TG2 were evaluated (Figure 3).

The median BMI was 33.1 kg/m^2^ (31.2–35.9). Most participants at baseline were female (92.6%), with a median age of 61.4 years, married (55.8%), retired (36.3%), or housewives (30.1%). The most prevalent diseases were high blood pressure and dyslipidemia (63.2% and 54.0%, respectively). Almost half of participants were in the maintenance stage of change for weight reduction (45.4%) and had high self-efficacy (76.1%) (Table 2).

When comparing CG and IG participants, it was found that the prevalence of women was lower in IG (89.7% vs. 95.3%, *p* < 0.001), as was the prevalence of high blood pressure (59.9% vs. 66.3%, *p* = 0.028) (Table 2).

The median time between the end of baseline data collection and the beginning of the intervention was 27.5 (21.0–35.0) days, with no difference between TGs [26.5 (21.0–35.0) and 27.5 (21.0–35.0); *p* = 0.868]. The median duration of the intervention at each PAS unit in the IG was 5.6 (5.6–5.8) months, with no differences between TG1 and TG2 [5.8 (5.6–5.8) and 5.6 (5.6–5.8); *p* = 0.276]. The activities were not carried out in three PAS units due to the small number of eligible individuals. TG1 was developed in 14 units and TG2 in 24 units. TG1 had 98 face-to-face meetings, and TG2 had 252 meetings. A total of 13,473 phone calls were made, and 1973 postcards were delivered.

**Figure 4:**
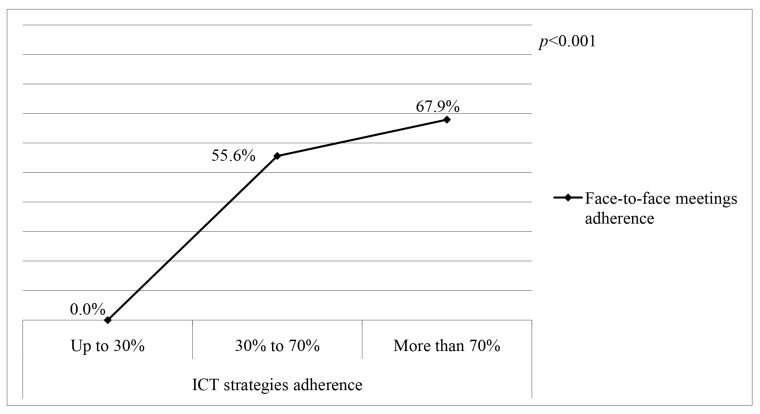
Comparison of the prevalence of adherence to face-to-face meetings between three ICT strategies adherence categories in the intervention group. Note: ICT = Information and Communication Technologies; *p* value: Kruskal–Wallis.

The median total adherence to the intervention was 52.4% (26.8–70.2), which was different between the TGs [TG1 = 47.8% (23.8–64.9) and TG2 = 56.9% (31.1–71.5); *p* = 0.002]. When considering face-to-face meetings, the median adherence was 45.7% (14.3–73.5), without difference between the groups [TG1 = 44.9% (14.3–71.4) and TG2 = 55.6% (11.1–77.8); *p* = 0.184]. The ICT strategies had a median adherence of 56.3% (31.2–80.0), with a difference between the groups [TG1 = 56.3% (25.0–56.3) and TG2 = 60.0% (40.0–80.0); *p* < 0.001]. Adherence to face-to-face meetings was associated with ICT strategies; the higher the adherence to ICT strategies (more than 70% vs. 30% to 70%), the higher the adherence to face-to-face meetings (67.9% vs. 55.6%) (*p* < 0.001) (Figure 4).

## 4. Discussion

The nutrition interventions were implemented as scheduled and lasted for the planned duration of activities. Adherence was approximately 50%, and the use of ICT strategies led to an increase in participation in face-to-face meetings. The participants were mostly women who were married, retired, or housewives, with self-reported high blood pressure or dyslipidemia, and in the maintenance stage of change for weight reduction.

The profile of the participants is similar to other studies in the PAS and PHC-based obesity management [25,26,30]. Since program activities typically take place during business hours, it is expected that most participants will be retired or housewives. Furthermore, users have access to PAS through referrals from health services, so it is common for them to have a history of comorbidities, such as hypertension or dyslipidemia [45,46]. In addition, as they are taking care of their health by exercising in a health promotion setting, many report being in the action or maintenance stages of change for various health-related behaviors.

The interventions were delivered within the proposed timeframe, with minor adjustments in schedules to accommodate weekends and holidays. Building the rapport with PAS teams before initiating the nutrition interventions was relevant for developing the optimal schedule for meetings in each PAS unit. The presence of multiple intervention team members at each group meeting was also feasible due to each team having a predefined team schedule and was a useful strategy for providing multidisciplinary obesity care.

As for the participants, a shorter interval between initial meetings facilitated rapport between the intervention team and participants in the first activities. Larger intervals between later meetings supported participants in their process of empowerment and autonomy for self-care [9,47]. This result is significant for PHC-based obesity management. As for the replication of nutritional interventions in other PHC services, the “Instructive of Collective Approach for the obesity management in SUS” was designed for health professionals to utilize, and as we demonstrate that the activities can be completed within 60 min and their methods are feasible, we reinforce the feasibility of the strategy outlined in the Instructive. The success of intervention delivery is crucial because multi-component interventions lasting up to six months can effectively lead to clinically meaningful weight loss in adults with obesity [48].

However, the median total adherence rate was lower than what was found in a meta-analysis with the aim of quantifying overall adherence rates for various weight loss interventions, which was 60.5% [49]. The lower adherence observed can be attributed to the post-COVID-19 period during which the group met, as health services were in the process of reopening. Additionally, the meeting times coincided with the PAS practice for some participants, which made it easier for them to attend. However, for others, having to attend at a different time posed challenges that made it difficult for them to leave.

Participants in TG2 showed higher adherence rates than those in TG1. Interventions carried out with more frequent meetings keep participants more motivated [49]. Another reason individuals may have adhered more to TG2 is the severity of obesity, indicated by the presence of comorbidities. When a person has more than one related health issue, they become more health-conscious and more motivated to engage in activities that can alleviate their symptoms and improve overall well-being [45,46]. Subsequently, multivariate analyses adjusted for age, sex, time in the program, self-reported disease, and self-efficacy will be performed to verify the highest adherence rate in TG2.

The adherence to ICT strategies was consistent with the literature [50]. Delivering ICT activities has an enormous potential to reach participants between face-to-face meetings, given the ubiquity of smartphones and their ease of use [51]. Furthermore, these strategies provide an opportunity for continued contact with the team, which can increase and sustain motivation, improve treatment adherence, and help achieve behavior change and body weight reduction [51,52]. This remote support played a vital role in enhancing adherence to face-to-face meetings. A meta-analysis concluded that both face-to-face interactions and ICT activities, such as phone calls, text messages, and electronic feedback, significantly improve the success of interventions [53]. Additionally, the technology has shown potential to enhance adherence to dietary, behavioral, and multi-component interventions [9,54]. In addition, the combination of postcards delivered by PAS teams and motivational phone calls or messages delivered by the intervention team highlighted the partnership between PHC professionals and the research team, which is likely to have influenced the success of ICT strategies in this trial and their association with face-to-face meeting attendance.

Our interventions’ limitations include the lack of blinding, because participants were aware of their randomization status. However, this issue is inherent to the nature of behavior change interventions. Nonetheless, we avoided contamination of the CG by considering the entire PAS units for randomization, instead of randomizing individuals within the same units.

Despite the large sample size and its representativeness, PAS participants are not completely comparable with the general population due to the predominance of women, individuals from more vulnerable social strata, and those aged 40 or over [30], as shown in our preliminary results. Therefore, these aspects must be carefully considered when extrapolating these results to different populations. However, the populations of PAS and of PHC also have these predominant characteristics, and as around 60% of the Brazilian population is enrolled in and depends on public PHC services, this study can provide important evidence for improving obesity management in this setting [55]. In 2021, over 4 million individuals with obesity were served by PHC services, among whom 624 thousand had severe obesity [56].

The differences in gender and high blood pressure between the IG and CG participants represent a limitation. However, the sample was randomized, and this difference appeared to be random. The higher percentage of women and the lower prevalence of high blood pressure in the IG may confound future analyses, so it is important to indicate that subsequent effectiveness analyses will be adjusted for these variables.

Not having performed activities in all PAS units due to the reduced number of eligible participants and needing to give up TG1 to focus on TG2 in some units are also limitations. These adjustments were necessary due to the low number of eligible users for TG1 in these units, which hindered the delivery of group meetings or forced the combination of TG1 and TG2 participants in the same group (61 participants across units).

This study also presents relevant potentials. Evaluating the effectiveness of the Brazilian Ministry of Health’s proposal for obesity treatment in PHC settings is a major strength of our study, which will provide evidence for programs and policies. This is necessary considering that Brazil has not reached the target of stopping the growth of obesity in adults proposed in the Strategic Actions Plan for Tackling Noncommunicable Diseases [57]. Another strength is the design, development, and evaluation of the interventions, which considered participants’ socioeconomic status and cultural context, life experiences, and readiness to reduce body weight [58]. Interventions were also based on person-centered approaches and current scientific evidence from a systematic review, which highlights the use of ICT to improve participant adherence and the relevance of a regressive frequency for face-to-face meetings.

Another strength of this study was the differentiation of nutrition interventions according to the severity of obesity. Individuals with more severe obesity tend to have more comorbidities and a longer history of unsuccessful body weight reduction attempts [59,60]. In this study, greater adherence to nutritional interventions was observed among participants with more severe disease compared to those with less severe disease. Therefore, obesity management interventions aimed at this group must be tailored to their specific needs to be effective and sustainable [61].

The proposed nutrition interventions were based on instructional materials focused on obesity management that can be utilized by all health professionals, not just dietitians, considering the structure of Brazilian PHC teams, which typically include physicians, nurses, nursing technicians, and community health workers. These materials are designed to be easy to understand and apply, providing practical, evidence-based guidelines that teams can incorporate into their daily routines while respecting the competencies of each professional category. This approach aims to enhance care capacity by promoting interdisciplinary collaboration, even in the absence of a complete multidisciplinary team. We believe that this proposal has the potential to be effectively applied in various contexts within PHC, including municipalities with fewer available specialists.

## 5. Conclusions

This study provides valuable insights into the feasibility, implementation, and impact of multi-component interventions for obesity management in the context of Brazilian PHC. The nutritional interventions were delivered as planned, with high fidelity to the proposed model, and demonstrated good feasibility within routine PHC settings. Despite some limitations, the results underscore the potential of structured, evidence-based programs tailored to the population’s needs.

Importantly, the interventions addressed the complex realities of PHC users by incorporating person-centered approaches and leveraging ICT strategies to maintain engagement and improve adherence. The design also accounted for different levels of obesity severity, offering tailored strategies that align with current evidence and best practices. Ultimately, this study strengthens the evidence base for the Brazilian Ministry of Health’s approach to obesity management, contributing to the development of scalable and culturally sensitive interventions capable of addressing the growing burden of obesity in the country. In the future, the effects of adherence on weight and waist circumference reduction, the progression of stages of change, and the eating behaviors of the studied population will be assessed.

## Figures and Tables

**Table 1:** Description of face-to-face meetings and ICT strategies employed in group-based nutrition interventions according to the treatment group.

Therapeutic Group	Meeting Strategy and Theme	Objectives	Activities	Materials	Methods
TG1 and TG2: Face-to-face meeting number 1	Workshop: What brings me here?	-Promote group integration -Reflect on everyday life and how it affects health -Recognize motivations and expectations related to participation in the group -Choose group name -Know readiness for change -Build coexistence contract	-Participants’ expressions about “What brings me here?” -Construction of collaborative text: expectations with the group -Presentation of the group’s proposal -Explanation of the stages of change -Construction of the Coexistence Contract	-Copies of the central question: “What brings me here?” -Pens, pencils, erasers and markers -Blank sheets and Kraft paper	-The team presented themselves. -A document was filled out addressing the guiding question: “What brought me here?”. Participants shared their likes and dislikes, joys and sorrows, a bit about their lives, and how all of that related to their health. -Based on the responses, the group collectively identified their expectations and motivations for participation in the TG. -An explanation of the stages of change was provided. -The group’s goals, characteristics, duration, and meeting frequency were clearly defined. -Key points that would support the group’s effective functioning were agreed upon and recorded in the Coexistence Contract.
TG2: Face-to-face meeting number 2	Workshop: How I would like to look	-Build self-image -Reflect on conflicts, desires, self-esteem, and the possibility of change -Recognize the dynamic nature of self-image	-Self-portrait productions	-Kraft paper and masking tape -Crayons, colored pencils and gouache paint -Painting brushes of different sizes -Sound speakers	-A moment of relaxation began with deep breathing exercises, focusing on bodily sensations. -Each participant was invited to represent their ideal body image (how they perceive themselves or how they wish to be). -Those who chose to present their self-portrait were asked to select at least one strategy to attain the health and body image they desired.
TG1: Face-to-face meeting number 2 TG2: Face-to-face meeting number 3	Workshop: Why reduce weight?	-Discuss the relationship between weight and health -Express aspects of daily life that promote or hinder weight reduction	-“Balance of Choices”	-Filled non-woven bags in different sizes -Adhesive tags -Pens and pencils -Handcrafted scale	-The conversation focused on the facilitators and hindrances to weight loss. -Each participant selected a bag to represent the factors that either facilitate or impede weight loss. Each bag was labeled with a corresponding factor, and they varied in weight to symbolize how some influences can be more significant than others. Participants chose bags based on which factors they felt had more or less impact on their weight loss journey. -The bags were placed on a two-pan scale, and participants were asked to reflect on which side was heavier: the facilitators or the hindrances. -The group discussed the importance of selecting dietary and physical practices to support weight loss goals. -Participants shared their experiences of successful changes and how these changes affected their lifestyles and health care, contributing to their sense of self-efficacy.
TG1: Face-to-face meeting number 3	Workshop: How do I know my body is hungry?	-Understand biological mechanisms of hunger and satiety -Make an analogy between these mechanisms and sensations of emptiness and fullness -Recognize that food fulfills biological and psychological needs through the symbolic meanings and pleasure they provide -Check strategies that feed the body and emotions	-Experience of mindful eating -Demonstration of the gastric emptying mechanism -Ways of eating and satiety	-Raisins -Small plastic bag -Funnel -Water and large water bottle -Marbles -Sound speakers -Copies of the “Hunger and satiety journal”	-The act of eating raisins was practiced with full attention. First, participants ate without focusing, and then they were taught to eat mindfully. They discussed the advantages of this mindful eating practice. -A demonstration of gastric emptying was conducted using a bag, marbles, and water. The discussion included the consumption of solids and liquids, as well as the concept of satiety. -Participants were provided with a hunger and satiety diary to fill out at home. Before starting a meal, they were instructed to record their level of hunger on a scale from 0 to 10, where 0 means “not hungry” and 10 means “most hungry.” After the meal, they noted which foods they ate and their level of satiety on the same scale, where 0 means “not at all satiated” and 10 means “fully satiated.”
TG1 and TG2: Face-to-face meeting number 4	Workshop: Myths and truths	-Identify myths and truths about reducing/maintaining a healthy weight -Clarify doubts about obesity treatment -Know the importance of seeking reliable information to treat obesity	-Game: “I agree, I disagree, I am unsure” -Demystifying doubts about weight reduction -Reliable sources of information	-Kraft paper -Colored pens -Adhesive tape and scissors -Folder “Reliable sources of information” -Dietary Guidelines for the Brazilian Population	-Participants wrote down their weight loss and maintenance strategies on posters, categorizing them under the themes “I agree,” “I disagree,” and “I have questions.” They used the guiding phrase, “For the treatment of obesity...” to structure their responses. -After collecting the information on the posters, similar themes were grouped together, and a discussion followed. -Folders containing suggestions for books and websites that participants could safely consult were distributed.
TG1 and TG2: Face-to-face meeting number 5	Workshop: Cooking: the basis of a healthy diet	-Build the concepts of fresh, minimally processed, processed and ultra-processed foods, and culinary ingredients -Recognize the act of cooking as a strategy for healthy weight reduction	-Degree of food processing -Culinary workshop	-Graphical representation of food processing degrees -Food replicas -Ultra-processed food labels -Recipe ingredients -Kitchen utensils -Copies of recipes -Folder on food classification -Folder on culinary skills	-The discussion focused on food choices and food processing, using a ladder representation to illustrate different food groups. Examples included milk (minimally processed), cheese (processed), a milk drink (ultra-processed), and olive oil (culinary ingredient). -Food labels were distributed to emphasize that ultra-processed foods often contain many ingredients, most of which may be unfamiliar. -Two recipes were prepared: a colorful salad and mint sauce. There was a discussion on culinary preparations, the use of culinary ingredients, and how to substitute ultra-processed foods with minimally processed options.
TG1 and TG2: Face-to-face meeting number 6	Workshop: Planning to hit the target	-Recognize the importance of planning for change -Define individual goals to reduce weight -Identify obstacles for weight reduction goals -Develop a positive attitude towards lapses and relapses	-Assembling puzzles with and without planning -Action plan	-Puzzle games -Printed image of the puzzle -Copies of the “Action Plan” -Pencil/pen and eraser	-Participants were grouped and invited to assemble a puzzle freely within a limited timeframe, without any prior planning. The expectation was that they would be unable to complete the puzzle in the allotted time. -After that, a new strategy was proposed: to assemble the puzzle by colors, starting with the edges and visualizing the image, while allowing for more time. -The discussion highlighted the importance of planning to achieve goals. -A document was distributed for participants to complete an action plan. This plan aimed to identify the desired change in habit, the start date, specific actions to be taken (such as frequency and timing), potential obstacles, and strategies to overcome those obstacles.
TG1: Face-to-face meeting number 7	Workshop: What do I take from here?	-Know the evolution of weight -Share achieved results -Value intermediate results -Strengthen the group as a social support strategy	-“What brings me here?” vs. “What do I take from here?”	-Copies with the central question: “What do I take from here?” -Pens, pencils and erasers -Texts from meeting number 1	-Participants were provided with a copies that invited them to reflect on their experiences in the group and how it impacted their lives and health. Additionally, the expectations set for the first meeting were revisited to determine whether they had been fulfilled.
TG2: Face-to-face meeting number 7	Workshop: Knowing food servings	-Discuss preparation adaptations to reduce quantities of culinary ingredients -Know serving sizes of foods sold and consumed -Discuss strategies to reduce the amounts of food consumed	-Home cooking and measurements -Portioning of culinary preparations -Tasting of the preparations Culinary	-Kitchen measures kit -Recipe ingredients -Salt and sugar -Kitchen utensils -Copies of recipes	-The meeting started with the preparation of recipes for a refreshing lettuce salad, avocado sauce, and a fruit juice blend of pineapple, orange, and apple. -During the preparation, we emphasized the importance of adapting culinary techniques and introduced a discussion about portion sizes. -We presented the most commonly used household measurements in cooking and food consumption. -Participants were served different portion sizes for comparison. -Finally, everyone had the opportunity to taste the prepared recipes.
TG2: Face-to-face meeting number 8	Workshop: Living with support	-Reflect on the responsibility of self-care -Propose solutions/strategies for barriers experienced in reducing weight -Recognize the importance of supported self-care -Reflect on the importance of preventing relapses -Motivate self-assessment of the change process	-Obstacles for weight reduction -Postcard construction	-Party balloons -Sound speakers -White paper -Clippings from old magazines -Pens, colored paints and pencils, crayons -Paper strips, colored ribbons, string and glue	-During the discussion, we addressed the challenges that can arise along the way and emphasized the importance of developing strategies to overcome them. We also highlighted the significance of seeking support to tackle these obstacles. -Each participant wrote down their individual challenges, and then we exchanged papers. Based on the discussions from previous meetings, everyone provided suggestions to help overcome each other’s obstacles. -To conclude the session, each participant created a postcard for their colleague, featuring the suggested strategies.
TG2: Face-to-face meeting number 9	Action on the environment: Sharing real food	-Understand the territory as a living space for food, leisure and pleasure -Value dining and real food	-Picnic	-Large tablecloth -Kitchen utensils -Healthy foods	-Participants were invited to bring food or culinary creations to share, inspired by authentic local cuisine. The offerings included a variety of dishes and preparation methods, such as cakes, pies, cookies, juices, and fruit salad. However, participants were instructed to avoid ultra-processed foods and to minimize the use of salt, oil, and sugar in their preparations. -Discussions took place about each dish presented, focusing on the lessons learned during the meetings.
TG1: ICT strategy number 1	Postcard: Your presence matters	-Re(know) topics covered -Feel motivated to participate in the group and the next meeting	-Postcard delivery	-Postcard	-Delivery of the postcard by the physical educator on the scheduled date.
TG1: ICT strategy number 2	Postcard: Gains and challenges along the way		-Postcard delivery	-Postcard	-Delivery of the postcard by the physical educator on the scheduled date.
TG1: ICT strategy number 3	Postcard: Importance of changing		-Postcard delivery	-Postcard	-Delivery of the postcard by the physical educator on the scheduled date.
TG1: ICT strategy number 4	Message or call: Our goals		-Motivational phone call or text message	-Cell phone	-The research team made telephone calls.
TG2: ICT strategy number 1	Message or call: You are important		-Motivational phone call or text message	-Cell phone	-The research team made telephone calls.
TG2: ICT strategy number 2	Postcard: Take care of yourself today and everyday		-Postcard delivery	-Postcard	-Delivery of the postcard by the physical educator on the scheduled date.
TG2: ICT strategy number 3	Postcard: “Health + flavor”		-Postcard delivery	-Postcard	-Delivery of the postcard by the physical educator on the scheduled date.
TG2: ICT strategy number 4	Message or call: Action plan underway		-Motivational phone call or text message	-Cell phone	-The research team made telephone calls.
TG2: ICT strategy number 5	Postcard: Gains and challenges along the way		-Postcard delivery	-Postcard	-Delivery of the postcard by the physical educator on the scheduled date.

Note: TG = Therapeutic Group; ICTs = Information and Communication Technologies.

**Table 2:** Demographics, health characteristics, and readiness for change of baseline eligible participants.

Variables	Total (*n* = 1120)		Control Group (*n* = 575)		Intervention Group (*n* = 545)		
	*n*	Value (95%CI or IR)	*n*	Value (95%CI or IR)	*n*	Value (95%CI or IR)	*p* Value
**Female gender** (%)	1037	92.6 (90.9–94.0)	548	95.3 (93.2–96.8)	489	89.7 (86.9–92.0)	<0.001
**Age** (years) (median)	1119	61.4 (53.8–67.1)	574	61.3 (53.8–67.0)	545	61.6 (53.9–67.3)	0.492 *
**Monthly per capita income** (R$) ^1^ (median)	988	1000 (606–1450)	511	1000 (606–1333)	477	1000 (606–1500)	0.946 *
**Education** (years) ^2^ (median)	1116	8 (4–11)	573	8 (4–11)	543	8 (4–11)	0.869 *
**Marital status** (%)							
Married	625	55.8 (52.9–58.7)	307	53.4 (49.3–57.4)	318	58.3 (54.1–62.4)	0.303
Single	185	16.5 (14.4–18.8)	100	17.4 (14.5–20.7)	85	15.6 (12.8–19.0)	
Divorced	131	11.7 (9.9–13.7)	75	13.0 (10.5–16.1)	56	10.3 (8.0–13.1)	
Widowed	179	16.0 (14.0–18.2)	93	16.2 (13.4–19.4)	86	15.8 (12.9–19.1)	
**Professional occupation** (%)							
Housewife	337	30.1 (27.5–32.8)	172	29.9 (26.3–33.8)	165	30.3 (26.5–34.3)	0.768
Retired	407	36.3 (33.6–39.2)	205	35.6 (31.8–39.7)	202	37.1 (33.1–41.2)	
Unemployed	48	4.3 (3.2–5.6)	28	4.9 (3.4–7.0)	20	3.7 (2.4–5.6)	
Employed	328	29.3 (26.7–32.0)	170	29.6 (26.0–33.4)	158	29.0 (25.3–32.9)	
**Self-reported diseases** (%)							
High blood pressure ^3^	707	63.2 (60.3–66.0)	381	66.3 (62.3–70.0)	326	59.9 (55.7–64.0)	0.028
Dyslipidemia ^4^	598	54.0 (51.0–56.9)	297	52.1 (48.0–56.2)	301	55.9 (51.7–60.1)	0.200
Joint disease	364	32.6 (29.9–35.4)	201	35.2 (31.4–39.2)	163	29.9 (26.2–33.9)	0.059
Diabetes	336	30.0 (27.4–32.8)	181	31.6 (27.9–35.5)	155	28.4 (24.8–32.4)	0.251
Cardiovascular disease	99	8.9 (7.3–10.7)	51	8.9 (6.8–11.5)	48	8.8 (6.7–11.5)	0.949
Sleep apnea ^5^	92	8.2 (6.8–10.0)	47	8.2 (6.2–10.8)	45	8.3 (6.2–10.9)	0.966
**Stage of change** (%)							
Preparation	169	15.1 (13.1–17.3)	80	14.0 (11.3–17.0)	89	16.3 (13.4–19.7)	0.543
Action	441	39.4 (36.6–42.3)	229	40.0 (36.0–44.0)	212	38.9 (34.9–43.1)	
Maintenance	508	45.4 (42.5–48.4)	264	46.1 (42.0–50.2)	244	44.8 (40.6–49.0)	
**Self-efficacy** (%) ^3^							
Reduced	267	23.9 (21.5–26.5)	148	25.8 (22.4–29.6)	119	21.9 (18.6–25.5)	0.121
High	850	76.1 (73.5–78.5)	425	74.2 (70.4–77.6)	425	78.1 (74.4–81.4)	

Note: Value = percentage or median; 95%CI = 95% Confidence Interval; IR = Interquartile Range; *p* value: Chi-Square test; * Mann–Whitney; ^1^ 68 missing; ^2^ 2 missing; ^3^ 1 missing; ^4^ 6 missing; ^5^ 2 missing.

## Data Availability

The anonymized datasets generated and/or analyzed during the current study will be available from the corresponding author upon reasonable request.

## Supplementary Materials

The supporting information can be downloaded at https://www.mdpi.com/article/10.3390/ijerph22071093/s1. Table S1: Number of participants in each district by Control and Intervention Group; Figure S1: Screening questionnaire; Figure S2: Baseline questionnaire; Figure S3: Reassessment questionnaire; Figure S4: Description of the ICT activities.
