# Supplementary material for: Guidelines and Preliminary Results of Group-Based Nutrition Interventions for Obesity Management Among Adults in Brazilian Primary Health Care
Source: Int J Environ Res Public Health. 2025 Jul 9;22(7):1093. doi: 10.3390/ijerph22071093 (PMC12294690; doi:10.3390/ijerph22071093)
Supplement: Supplementary file 1 [file ijerph-22-01093-s001.zip › ijerph-3644783-supplementary.pdf]

Suplementary Material

Table S1: Number of participants in each district by Control and Intervention Group

District	PAS unit (participants)	
	Control Group	Intervention Group
Barreiro	Bairro das Indústrias (n=16)	Diamante (n=34)
	Petrópolis (n=23)	Parque das Águas (n=35)
	Milionários (n=22)	Vale do Jatobá (n=23)
	Vila Pinho (n=26)	Regina (n=27)
	Vila Santa Rita (n=20)	
	Lindeia (n=36)	
Centro Sul	Santa Rita de Cássia (n=17)	Santa Lúcia (n=7)
	Tia Amância (n=1)	Oswaldo Cruz (n=5)
	Laboratório do Movimento (n=15)	
Leste	Mariano de Abreu (n=19)	Sagrada Família (n=18)
	Boa Vista (n=18)	Paraíso (n=7)
	Riviera (n=30)	São Geraldo (n=24)
Nordeste	Dom Joaquim (n=21)	Jardim Belmonte (n=23)
	Ribeiro de Abreu (n=9)	Jardim Vitória (n=20)
	União (n=12)	Paulo VI (n=30)
	Santa Cruz (n=27)	
Noroeste	Centro de Referência do Idoso (n=7)	Fazendinha (n=6)
	CIAME (n=6)	Califórnia (n=7)
	Coqueiral (n=15)	Ermelinda (n=20)
		Mercado da Lagoinha (n=29)
Norte	Zilah Spósito (n=10)	Monte Azul (n=14)

	Campo Alegre (n=19)	Via 240 (n=45)
	Vila Biquinhas (n=28)	Providência (n=19)
	Jardim Felicidade (n=26)	
	Conjunto Betânia (n=7)	Palmeiras (UNI-BH) (n=16)
Oeste	Havaí (n=9)	CRAS Vista Alegre (n=18)
	Vila Leonina (n=20)	Ventosa (n=15)
	São Francisco (n=12)	Confisco (n=25)
Pampulha	São José (n=19)	Universitário (n=24)
	Serrano (n=19)	
	Céu Azul (n=13)	Santa Mônica (n=21)
Venda Nova	Lagoa (n=20)	Jardim Leblon (n=18)
	Serra Verde (n=33)	Venda Nova (n=15)

Figure S1: Screening questionnaire

UFMG

Qualificação do manejo da obesidade no SUS: efetividade de intervenção coletiva
Avaliação da efetividade de intervenção coletiva para o manejo da obesidade em usuários da Atenção Primária com indicação de cirurgia bariátrica

CUIDADO DA PESSOA COM OBESIDADE NO SUS

Data entry - Date: _____/_____/20____ Name: ____
Interviewer: Leave it to the field supervisor to fill in.

INSTRUMENT OF SCREENING

INTERVIEWER: PLEASE, FILL IN THE QUESTIONNAIRE IN PENCIL, WITH LETTER IN A LEGIBLE FORM.
COMPLETE QUESTIONS 1 AND 2 BEFORE STARTING THE INTERVIEW.

1. Interviewer: ____

2. Date of interview: _____/_____/20____ 3. Start time: _____:_____

4. Full Name:____

5. Number of identification: _______ *Interviewer: leave for the supervisor of field to fill in.*

6. Location: *Interviewer: no ask; just register the unit from the Academy from the City (PAC).*

(0) Diamante	(1) Parque das Águas	(2) Vale do Jatobá	(3) Regina
(4) Bairro Indústrias	(5) Petrópolis	(6) Milionários	(7) Vila Pinho
(8) Vila Santa Rita	(9) Lindéia	(10) Santa Lúcia	(11) Oswaldo Cruz
(12) Santa Rita Cássia	(13) Tia Amância	(14) Lab. Movimento	(15) Sagrada Família
(16) Paraíso	(17) São Geraldo	(18) Mariano de Abreu	(19) Boa Vista
(20) Riviera	(21) Jardim Belmonte	(22) Jardim Vitória	(23) São Marcos
(24) Dom Joaquim	(25) Ribeiro de Abreu	(26) União	(27) Santa Cruz
(28) Fazendinha	(29) Califórnia	(30) Ermelinda	(31) Mercado Lagoinha
(32) Centro Ref. Idoso	(33) CIAME	(34) Coqueiral	(35) Monte Azul
(36) Providência	(37) Via 240	(38) Zilah Spósito	(39) Campo Alegre
(40) Vila Biquinhas	(41) Jardim Felicidade	(42) Palmeiras (Uni BH)	(43) CRAS Vista Alegre
(44) Ventosa	(45) Conjunto Betânia	(46) Havaí	(47) Vila Leonina
(48) Confisco	(49) Jardim Alvorada	(50) São Francisco	(51) São José
(52) Serrano	(53) Santa Mônica	(54) Jardim Leblon	(55) Venda Nova
(56) Céu Azul	(57) Lagoa	(58) Serra Verde	

7. What day(s) do you work out at the city gym? *Interviewer: mark all the options reported.*
(0) Monday (1) Tuesday (2) Wednesday (3) Thursday (4) Friday (5) Saturday

8. What time do you work out at the city gym?
(0) 07:00 (1) 08:00 (2) 09:00 (3) 10:00 (4) 11:00

I) SOCIODEMOGRAPHIC PROFILE

I.1. Address: ____ I.2. Postal Code: ______________

I.3. Telephone: ( ) ____________________ I.4. Cell phone: ( ) _____________________________

I.5. Sex: *Interviewer: no do this question; just check an option.* (0) Feminine (1) Masculine

I.6. Date of birth: _____/_____/_____

I.7. What is your marital status?
(0) Married/Common-law marriage (1) Single (2) Separated/Divorced (3) Widower

I.8. Up to what grade did you study? _________ years of study. *Interviewer: consult the manual to see how many years of study correspond to each grade.*

I.9. What is the monthly family income? R$______________ (77) Don't know (88) No if apply
Minimum wage=R$ 1,212.00

I.10. How many people live in your home? ___________________

I.11. What is your main occupation? *Interviewer: consider the occupation that generates the highest income.*

(0) Housewife (1) Retired (2) Unemployed (3) Other: ____________

I.12. Are you pregnant? *Interviewer: for men, do not ask this question; just mark 'No if apply'.*
(0) No (1) Yes (77) Don't know (88) No if apply

II) GROUP STRATIFICATION FOR OBESITY MANAGEMENT

Part 1 – Anthropometric measurement

II.1. Weight: __________kg II.2. Height: _________m II.3. BMI: _________kg/m²
Interviewer: calculate BMI=weight/height²
(99) No answer (99) No answer (88) No if apply

II.4. BMI classification (WHO, 2000): *Interviewer: just check one option according to II.3.*
(0) Low weight (BMI < 18.5 kg/m²) (1) Eutrophy (18.5 ≤ BMI ≤ 24.9 kg/m²)
(2) Overweight (25.0 ≤ BMI ≤ 29.9 kg/m²) (3) Obesity (BMI ≥ 30.0 kg/m²) (88) No if apply

II.5. Waist Circumference (WC):
1st measure: _____cm 2nd measure: _____cm 3rd measure: _____cm Average: _____cm

II.6. WC Classification – Metabolic risk (WHO, 2000): *Interviewer: check one option according to the sex (I.5) of the participant.*
Feminine: (0) WC ≥ 80 and < 88 cm (high risk) (1) WC ≥ 88 cm (very high risk) (88) No if apply
Masculine: (0) WC ≥ 94 and < 102 cm (high risk) (1) WC ≥ 102 cm (very high risk) (88) No if apply

Interviewer: If the user (non-pregnant) is identified as obese, continue. If not, end the collection, read the text below about the participant's NON-eligibility and mark "no if apply" in the next questions of the screening instrument.

**NO SELECTED (NOT ELIGIBLE)**	*"(Participant's name), thank you for participating in the research. In the next stages, we will only interview participants with obesity and other specific characteristics. After seeing your answers, you were not considered eligible to continue the research. But don't worry. You will continue to participate normally in the routine activities of Academia da Cidade and we are here to clarify any questions you may have about food and nutrition. Thank you very much for answering this questionnaire and contributing to science and public health!"*

Part 2: Indication for bariatric surgery

II.7. Have you ever had obesity surgery (bariatric surgery)? *Interviewer: if no, jump to question II.8.*
(0) No (1) Yes (77) Don't know (88) No if apply (99) No answer

II.7.1. If so, how long ago? ________years (88) No if apply

II.7.2. Did you regain weight after surgery?
(0) No (1) Yes (77) Don't know (88) No if apply (99) No answer

II.8. Are you currently awaiting bariatric surgery?
(0) No (1) Yes (77) Don't know (88) No if apply (99) No answer

II.9. Has a doctor ever diagnosed you with...

II.9.1. Cardiovascular disease (ex.: coronary artery disease, coronary heart disease, congestive heart failure, stroke)?
(0) No (1) Yes (77) Don't know (88) No if apply (99) No answer

II.9.2. High cholesterol and/or triglycerides?
(0) No (1) Yes (77) Don't know (88) No if apply (99) No answer

II.9.3. Sleep apnea (pauses in breathing while sleeping)?
(0) No (1) Yes (77) Don't know (88) No if apply (99) No answer

II.9.4. Joint disease/Osteoarthritis?
(0) No (1) Yes (77) Don't know (88) No if apply (99) No answer

II.9.5. Diabetes?
(0) No (1) Yes (77) Don't know (88) No if apply (99) No answer

II.9.6. High blood pressure?
(0) No (1) Yes (77) Don't know (88) No if apply (99) No answer

II.10. Do you currently smoke any tobacco products? *Interviewer: consider the following examples: cigarettes, straw cigarettes, clove or Bali cigarettes, pipes, cigarillos, hookah, etc.*

(0) Not currently smoke (1) Yes, less than daily (2) Yes, daily (88) No if apply (99) No answer

Part 3: Desire and time available for groups

II.11. Do you want to participate in obesity treatment groups lasting 6 months at City Academy?
(0) No (1) Yes (88) No if apply (99) No answer

II.12. Do you have time to participate in one of these groups here at City Academy?
(0) No (1) Yes (88) No if apply (99) No answer

Interviewer: even if the user answers 'No' to II.11 or II.12, continue. At the end, read the text about the participant's NON-eligibility.

Parte 4: Stage of change, self-efficacy and decision balance for weight reduction

II.13. Identifying the stage of change to reduce weight. *Interviewer: ask the relevant questions, and at the end check ONLY ONE STAGE.*

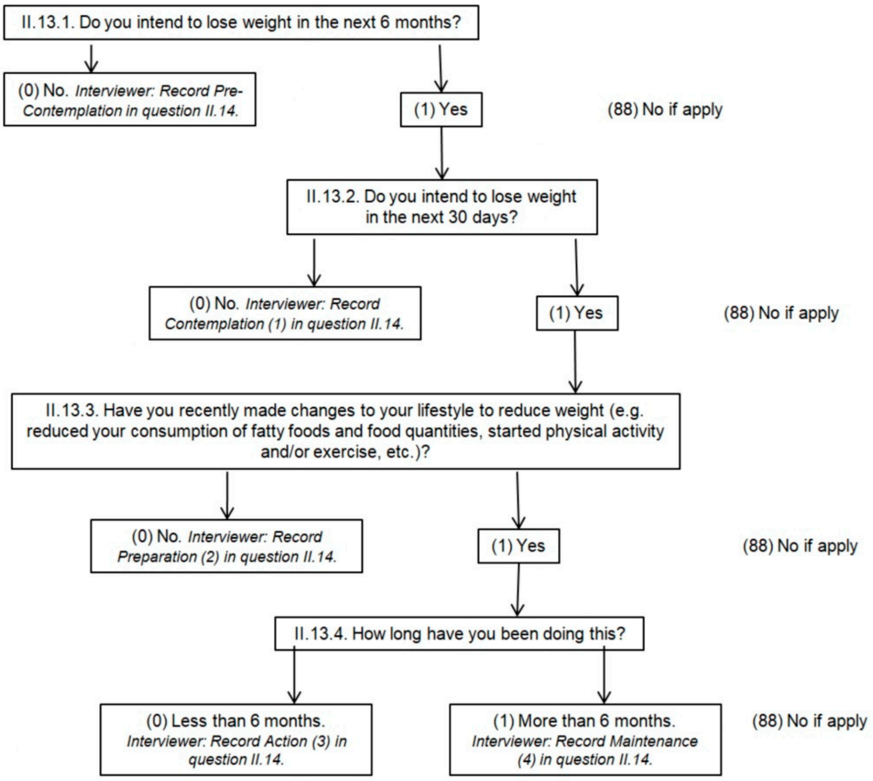

II.14. Result: *Interviewer: don't ask this question; just check one option.*
(0) Pre-Contemplation (1) Contemplation (2) Preparation (3) Action (4) Maintenance (88) No if apply

Interviewer: If pre-contemplation or contemplation, end the collection, read the text below about the participant's NON-eligibility and mark "not applicable" in the next questions of the screening instrument.

**NO SELECTED (NOT ELIGIBLE)**	*"(Participant's name), thank you for participating in the research. In the next stages, we will only interview participants with obesity and other specific characteristics. After seeing your answers, you were not considered eligible to continue the research. But don't worry. You will continue to participate normally in the routine activities of Academia da Cidade and we are here to clarify any questions you may have about food and nutrition. Thank you very much for answering this questionnaire and contributing to science and public health!"*

II.15. Identification of self-efficacy to reduce weight.

How confident are you that you can lose weight even if... *Interviewer: read the answer options for each situation. Use the form with the scale printed on it.*

II.15.1. ...need a lot of time to improve your practices?	(1) Not at all confident	(2) Somewhat confident	(3) Moderately confident	(4) Very confident	(5) Completely confident	(88) No if apply	(99) No answer
II.15.2. ...need many attempts until you succeed?	(1) Not at all confident	(2) Somewhat confident	(3) Moderately confident	(4) Very confident	(5) Completely confident	(88) No if apply	(99) No answer
II.15.3. ...need to rethink your strategies?	(1) Not at all confident	(2) Somewhat confident	(3) Moderately confident	(4) Very confident	(5) Completely confident	(88) No if apply	(99) No answer

II.16. Result. *Interviewer: don't ask this question; just check one option.*
(0) Low self-efficacy (1) High self-efficacy **(At least 2 answers (4) or (5) in II.15)** (88) No if apply

II. 17. Identifying the balance of decisions to reduce weight.

Indicate how important each of the situations below is to you at the moment. *Interviewer: read the answer options for each situation. Use the form with the printed scale.*

II.17.1. Plan more carefully when buying and/or preparing food to contribute to weight loss.	(1) Not at all important	(2) Slightly important	(3) Important	(4) Very important	(88) No if apply	(99) No answer
II.17.2. Feel good about taking care of your health during the weight loss process.	(1) Not at all important	(2) Slightly important	(3) Important	(4) Very important	(88) No if apply	(99) No answer
II.17.3. Learn to deal with frustrations that arise during the weight loss process.	(1) Not at all important	(2) Slightly important	(3) Important	(4) Very important	(88) No if apply	(99) No answer
II.17.4. Be able to identify the situations in your daily life that hinder weight loss.	(1) Not at all important	(2) Slightly important	(3) Important	(4) Very important	(88) No if apply	(99) No answer

Indicate how worrying each of the situations below is for you at the moment. *Interviewer: Read the response options for each situation. Use the form with the printed scale.*

II.17.5. Returning to some behavior that hinders weight loss.	(1) Not at all worrying	(2) Slightly worrying	(3) Worrying	(4) Very worrying	(88) No if apply	(99) No answer
II.17.6. Feeling unsupported in the weight loss process.	(1) Not at all worrying	(2) Slightly worrying	(3) Worrying	(4) Very worrying	(88) No if apply	(99) No answer
II.17.7. Having little time available to include in your daily routine the changes necessary for weight loss.	(1) Not at all worrying	(2) Slightly worrying	(3) Worrying	(4) Very worrying	(88) No if apply	(99) No answer
II.17.8. Being discouraged by family and/or friends when starting a new attempt to lose weight.	(1) Not at all worrying	(2) Slightly worrying	(3) Worrying	(4) Very worrying	(88) No if apply	(99) No answer

Interviewer, read the result the follow second the eligibility of participant.

**NO SELECTED (NOT ELIGIBLE)**	*"(Participant's name), thank you for participating in the research. In the next stages, we will only interview participants with obesity and other specific characteristics. After seeing your answers, you were not considered eligible to continue the research. But don't worry. You will continue to participate normally in the routine activities of Academia da Cidade and we are here to clarify any questions you may have about*

	food and nutrition. Thank you very much for answering this questionnaire and contributing to science and public health!"
**SELECTED (ELIGIBLE)**	*"(Participant's name), thank you for participating in this stage of the research. After reviewing your answers, we have identified that you will be able to participate in group educational activities to treat obesity that will take place at this City Academy. The research team will contact you to inform you about the start of the activities. Thank you very much for answering this questionnaire and contributing to science and public health!"*

VIII) OBSERVATIONS

____
____

VIII.1. End time: _____:_____

Interviewer, before releasing the user, check that all questions have been filled out correctly.

Figure S2. Baseline questionnaire

UFMG

Qualificação do manejo da obesidade no SUS: efetividade de intervenção coletiva
Avaliação da efetividade de intervenção coletiva para o manejo da obesidade em usuários da Atenção Primária com indicação de cirurgia bariátrica

CUIDADO DA PESSOA COM OBESIDADE NO SUS

Data entry - Date: _____/_____/20____ Name: ____
Interviewer: Leave it to the field supervisor to fill in.

BASELINE INSTRUMENT – TG1 and TG2

INTERVIEWER: PLEASE, FILL IN THE QUESTIONNAIRE IN PENCIL, WITH LETTER IN A LEGIBLE FORM.
COMPLETE QUESTIONS 1 AND 2 BEFORE STARTING THE INTERVIEW.

1. Interviewer: ____

2. Date of interview: _____/_____/20____ 3. Start time: _____:_____

4. Full Name:____

5. Number of identification: _______ *Interviewer: leave for the supervisor of field to fill in.*

6. Location: *Interviewer: no ask; just register the unit from the Academy from the City (PAC).*

(0) Diamante	(1) Parque das Águas	(2) Vale do Jatobá	(3) Regina
(4) Bairro Indústrias	(5) Petrópolis	(6) Milionários	(7) Vila Pinho
(8) Vila Santa Rita	(9) Lindéia	(10) Santa Lúcia	(11) Oswaldo Cruz
(12) Santa Rita Cássia	(13) Tia Amância	(14) Lab. Movimento	(15) Sagrada Família
(16) Paraíso	(17) São Geraldo	(18) Mariano de Abreu	(19) Boa Vista
(20) Riviera	(21) Jardim Belmonte	(22) Jardim Vitória	(23) São Marcos
(24) Dom Joaquim	(25) Ribeiro de Abreu	(26) União	(27) Santa Cruz
(28) Fazendinha	(29) Califórnia	(30) Ermelinda	(31) Mercado Lagoinha
(32) Centro Ref. Idoso	(33) CIAME	(34) Coqueiral	(35) Monte Azul
(36) Providência	(37) Via 240	(38) Zilah Spósito	(39) Campo Alegre
(40) Vila Biquinhas	(41) Jardim Felicidade	(42) Palmeiras (Uni BH)	(43) CRAS Vista Alegre
(44) Ventosa	(45) Conjunto Betânia	(46) Havaí	(47) Vila Leonina
(48) Confisco	(49) Jardim Alvorada	(50) São Francisco	(51) São José
(52) Serrano	(53) Santa Mônica	(54) Jardim Leblon	(55) Venda Nova
(56) Céu Azul	(57) Lagoa	(58) Serra Verde	

7. What day(s) do you work out at the city gym? *Interviewer: mark all the options reported.*
(0) Monday (1) Tuesday (2) Wednesday (3) Thursday (4) Friday (5) Saturday

8. What time do you work out at the city gym?
(0) 07:00 (1) 08:00 (2) 09:00 (3) 10:00 (4) 11:00

9. The interviewed and control/intervention of which group? (0) TG1 (1) TG2
Interviewer: supervisor of field will fill the field.

I) HEALTH LITERACY

I.1. Do you have difficulty understanding the instructions given by your doctor or other health professional?
(1) A lot of difficulty (2) Some difficulty (3) No difficulty (99) No answer

II) EATING HABITS

II.1. Normally, which meals you performs during the day: *Interviewer: ask each snack.*

II.1.1. Coffee from the morning?	(0) No	(1) Yes	(99) No answer
II.1.2. Snack from the morning?	(0) No	(1) Yes	(99) No answer
II.1.3. Lunch?	(0) No	(1) Yes	(99) No answer
II.1.4. Snack from the late?	(0) No	(1) Yes	(99) No answer
II.1.5. To have lunch or snack from the night?	(0) No	(1) Yes	(99) No answer

II.1.6. Supper or snack before of sleep?	(0) No	(1) Yes	(99) No answer

II.2. Number of meals in a typical day: _______ *Interviewer: do not ask; add up the answers above.*

Now I'm going to ask you some questions about your eating habits of certain foods. Always consider the average consumption of these foods.

II.3. In how many days from the week the Mr/Mrs. usually eat fruits.
______days Interviewer: *if response for five or more days a week, ask II.3.1*
(0) Never or less of one once a week (99) No answer

II.3.1. In general, how many times a day the Mr/Mrs. eat fruits?
(0) One time a day (1) Two times a day (2) Three times or more a day (88) No if apply

II.4. How many days a week do you usually eat at least one type of vegetable *(without count potatoes, cassava, taro or yam)* as lettuce, tomato, cabbage, carrot, chayote, eggplant, zucchini?
_______days *Interviewer: if response for five or more days a week, ask II.4.1.*
(0) Never or less of one once a week (99) No answer

II.4.1. In general, how many times a day the Mr/Mrs. usually eat that type of vegetable?
(0) One time a day (1) Two times a day (2) Three times or more a day (88) No if apply

II.5. In a day common, how many spoons (soup) of vegetables do you eat?
______spoons/day (88) No if apply (99) No answer

II.5.1. Mode of preparation: (0) Raw (1) Sautéed/Cooked (88) No if apply (99) No asnwer

II.6. In a day common, how many spoons (soup) of vegetables do you eat?
______spoons/day (88) No if apply (99) No answer

II.6.1. Mode of preparation: (0) Raw (1) Sautéed/Cooked (88) No if apply (99) No asnwer

II.7. In how many days from the week the Mr/Mrs. usually eat bean?
______days (0) Never or less of one once a week (99) No answer

II.8. In how many days from the week, the Mr/Mrs. usually eat meat red (ox, pork, kid, goat, sheep, etc.)?
______days (0) Never or less of one once a week (99) No answer

II.9. In how many days from the week the Mr/Mrs. usually eat chicken/hen?
______days (0) Never or less of one once a week (99) No answer

II.10. In how many days from the week the Mr/Mrs. usually eat fish?
______days (0) Never or less of one once a week (99) No answer

II.11. In how many days from the week the Mr/Mrs. usually take juice of box/tin or soft drink in dust?
______days (0) Never or less of one once a week (99) No answer

II.12. In how many days from the week the Mr/Mrs. usually take refrigerator?
______days (0) Never or less of one once a week (99) No answer

II.13. In how many days from the week the Mr/Mrs. usually take milk? (of animal origin: cow, goat, buffalo, etc.)
______days (0) Never or less of one once a week (99) No answer

II.14. In how many days from the week the Mr/Mrs. usually eat food candy as stuffed biscuits/cookies, chocolate, jelly, candy and others?
______days (0) Never or less of one once a week (99) No answer

II.15. In how many days from the week the Mr/Mrs. usually eat drink chocolate milk or yogurt with flavor?
______days (0) Never or less of one once a week (99) No answer

II.16. In how many days from the week the Mr/Mrs. usually eat food as sausage, sausage, mortadella or ham?
______days (0) Never or less of one once a week (99) No answer

II.17. In how many days from the week the Mr/Mrs. usually eat food as bread of form, hot dog or hamburger?
______days (0) Never or less of one once a week (99) No answer

II.18. How many days last 1 kg of salt in the your home? ______days (99) No answer

II.18.1. How many people live in your household? __________
Interviewer: leave to the supervisor field to fill in. Supervisor: consult the Screening questionnaire.

II.19. How much sugar do you use in one month? _______ kg (99) No answer
Interviewer: If don't buy sugar, put zero.

II.19.1. How many people live in your household? __________
Interviewer: leave to the supervisor field to fill in. Supervisor: consult the Screening questionnaire.

II.20. What type of fat and used, *with bigger frequency*, in your home to sauté, fry or to roast the food?
Interviewer: Please check only one option.
(0) Oil olive (1) Oil vegetable (2) Butter (3) Margarine/cream/shortening vegetable (4) Lard/fat animal
(5) No use fat (6) Others: ___________ (99) No answer

II.20.1. Amount monthly from the fat reported: __________ *Interviewer: remember what one bottle of oil he has 900mL*
(88) No if apply (99) No asnwer

II.20.2. Number of people who consume fat: _________ *Interviewer: Pay attention to whether there are people who only have one meal at home and the possible differences in the number of people who eat at home during the weekend.*
(88) No if apply (99) No asnwer

III) REASONS FOR FOOD CHOICES. *Interviewer: Use the form with the scale printed on it.*

I eat what I eat, ...

**III.1. Preference**					
III.1.1 ... because I have willing of eat.	(1) Never	(2) Rarely	(3) To the times	(4) Often	(5) Always
III.1.2 ... because is yummy.	(1) Never	(2) Rarely	(3) To the times	(4) Often	(5) Always
III.1.3 ... because I like it.	(1) Never	(2) Rarely	(3) To the times	(4) Often	(5) Always
**III.2. Habits**					
III.1.1 ... because I am accustomed the eat that.	(1) Never	(2) Rarely	(3) To the times	(4) Often	(5) Always
III.1.2 ... because is what I generally eat.	(1) Never	(2) Rarely	(3) To the times	(4) Often	(5) Always
III.1.3. ... because I know the product.	(1) Never	(2) Rarely	(3) To the times	(4) Often	(5) Always
**III.3. Need and hunger**					
III.3.1. ... because I need energy.	(1) Never	(2) Rarely	(3) To the times	(4) Often	(5) Always
III.3.2. ... because it satisfies my hunger in a pleasant way.	(1) Never	(2) Rarely	(3) To the times	(4) Often	(5) Always
III.3.3. ...because I have hunger.	(1) Never	(2) Rarely	(3) To the times	(4) Often	(5) Always
**III.4. Health**					
III.4.1. ... for to maintain food balanced.	(1) Never	(2) Rarely	(3) To the times	(4) Often	(5) Always
III.4.2. ... because is healthy.	(1) Never	(2) Rarely	(3) To the times	(4) Often	(5) Always
III.4.3. ... because it keeps me energized and motivated.	(1) Never	(2) Rarely	(3) To the times	(4) Often	(5) Always
**III.5. Convenience**					
III.5.1. ... because is fast to prepare.	(1) Never	(2) Rarely	(3) To the times	(4) Often	(5) Always
III.5.2. ... because is convenient.	(1) Never	(2) Rarely	(3) To the times	(4) Often	(5) Always
III.5.3. ... because is easy to prepare.	(1) Never	(2) Rarely	(3) To the times	(4) Often	(5) Always
**III.6. Pleasure**					
III.6.1. ... because it gives me pleasure.	(1) Never	(2) Rarely	(3) To the times	(4) Often	(5) Always
III.6.2. ... for me give something really special.	(1) Never	(2) Rarely	(3) To the times	(4) Often	(5) Always
III.6.3. ... to me reward.	(1) Never	(2) Rarely	(3) To the times	(4) Often	(5) Always
**III.7. Traditional food**					
III.7.1. ... because belongs the right	(1) Never	(2) Rarely	(3) To the times	(4) Often	(5) Always

situations.					
III.7.2. ... by tradition (ex.: tradition of family, special occasions)	(1) Never	(2) Rarely	(3) To the times	(4) Often	(5) Always
III.7.3. ... because I grew up eating like this.	(1) Never	(2) Rarely	(3) To the times	(4) Often	(5) Always
**III.8. Natural questions**					
III.8.1. ... because is natural.	(1) Never	(2) Rarely	(3) To the times	(4) Often	(5) Always
III.8.2. ... because no contains harmful substances (e.g. pesticides, pollutants, antibiotics).	(1) Never	(2) Rarely	(3) To the times	(4) Often	(5) Always
III.8.3. ... because is organic.	(1) Never	(2) Rarely	(3) To the times	(4) Often	(5) Always
**III.9. Socialization**					
III.9.1. ... because it is part of a social situation.	(1) Never	(2) Rarely	(3) To the times	(4) Often	(5) Always
III.9.2. ... to what I may to spend time with other people.	(1) Never	(2) Rarely	(3) To the times	(4) Often	(5) Always
III.9.3. ... because social meetings become more enjoyable.	(1) Never	(2) Rarely	(3) To the times	(4) Often	(5) Always
**III.10. Price**					
III.10.1. ... because is cheap.	(1) Never	(2) Rarely	(3) To the times	(4) Often	(5) Always
III.10.2. ... because I don't want to spend a lot of money.	(1) Never	(2) Rarely	(3) To the times	(4) Often	(5) Always
III.10.3. ... because it is in promotion.	(1) Never	(2) Rarely	(3) To the times	(4) Often	(5) Always
**III.11. Visual attraction**					
III.11.1. ... because the presentation is attractive (e.g. packaging).	(1) Never	(2) Rarely	(3) To the times	(4) Often	(5) Always
III.11.2. ... because it immediately catches my attention (presentation in the supermarket, it's colorful).	(1) Never	(2) Rarely	(3) To the times	(4) Often	(5) Always
III.11.3. ... because I recognize from advertisements or I've seen it on TV.	(1) Never	(2) Rarely	(3) To the times	(4) Often	(5) Always
**III.12. Weight control**					
III.12.1. ... because has few calories.	(1) Never	(2) Rarely	(3) To the times	(4) Often	(5) Always
III.12.2. ... because I control my weight.	(1) Never	(2) Rarely	(3) To the times	(4) Often	(5) Always
III.12.3. ... because has little fat.	(1) Never	(2) Rarely	(3) To the times	(4) Often	(5) Always
**III.13. Emotion control**					
III.13.1. ... because I am sad.	(1) Never	(2) Rarely	(3) To the times	(4) Often	(5) Always
III.13.2. ... because I am frustrated.	(1) Never	(2) Rarely	(3) To the times	(4) Often	(5) Always
III.13.3. ... because I feel alone.	(1) Never	(2) Rarely	(3) To the times	(4) Often	(5) Always
**III.14. Social standards**					
III.14.1. ... because it would be impolite not to eat.	(1) Never	(2) Rarely	(3) To the times	(4) Often	(5) Always
III.14.2. ... for avoid disappoint somebody what is trying to please me.	(1) Never	(2) Rarely	(3) To the times	(4) Often	(5) Always
III.14.3. ... because I have to eat.	(1) Never	(2) Rarely	(3) To the times	(4) Often	(5) Always
**III.15. Social image**					
III.15.1. ... because it's in fashion.	(1) Never	(2) Rarely	(3) To the times	(4) Often	(5) Always

III.15.2. ... because it makes me project a good image to others.	(1) Never	(2) Rarely	(3) To the times	(4) Often	(5) Always
III.15.3. ... because others like it.	(1) Never	(2) Rarely	(3) To the times	(4) Often	(5) Always

V) HEALTH PROFILE

V.1. Has a doctor ever diagnosed you with liver disease (non-alcoholic fatty liver disease)?
(0) No (1) Yes (77) Don't know (99) No answer

V.2. At the moment, you receives treatment to depression, anxiety or other illness mental?
(0) No (1) Yes (77) Don't know (99) No answer

V.3. In general, as you evaluates the your health? *Interviewer: read to the alternatives.*
(1) Very bad (2) Bad (3) Regular (4) Good (5) Very good (99) No answer

V.4. How many days a week you usually to practice exercise physical or sport (e.g.: walking, running, weight training, water aerobics, stretching, pilates, team games, dancing, etc.) ? __________ days (99) No answer

V.4.1. On the day you do physical exercise or sport, how long does this activity last?
________ minutes (88) No if apply

V.5. What was your body weight 1 year ago (in kg)?
________ kg (77) Don't know (99) No answer

V.6. What was the heaviest weight you've ever had in your life? *Interviewer: to women, NO consider weight during pregnancy.*
________ kg (77) Don't know (99) No answer

V.6.1. How old were you?
________ years (88) No if apply

V.7. Would you like to weigh... *Interviewer: read the alternatives.*
(1) More (2) Less (3) Equal (77) Don't know (99) No answer

V.8. How often do you weigh yourself?
(0) Every day (including saturday and sunday) (1) 2-3 days per week (2) 1 day per week (3) Once every 2 weeks (4) Once a month (5) Once every 2 months (6) A few times a year (7) Never (77) Don't know (99) No answer

V.9. Have you ever tried to lose weight? *Interviewer: If no, jump to question V.10.*
(0) No (1) Yes (77) Don't know (99) No answer

V.9.1. How long were you able to maintain your weight loss?
(1) less than 3 months (2) 3-6 months (3) 7-12 months (4) 1-3 years (5) 3-5 years (6) more than 6 years (77) Don't know (88) No if apply (99) No answer

V.10. Have you tried to lose weight in the last year? *Interviewer: If no, jump to question V.11*
(0) No (1) Yes (77) Don't know (99) No answer

V.10.1. If so, how did you try to lose weight? *Interviewer: check all the reported options*
(0) Eating less food (1) Eating less caloric food (2) Eating less fat (3) Eating less carbohydrates (4) Exercising (5) Skipping meals/fasting (6) Using diet products (7) Using a liquid diet (8) With a weight loss program (9) Following a special diet (Dr. Atkins, lowcarb...) (10) With medication prescribed by a doctor (11) With over-the-countermedications, herbs or supplements (12) Starting or returning to smoking (13) With laxatives or vomiting (14) Having surgery, such as gastric Bypass (15) Drinking lots of water (16) Eating more fruits, vegetables (salads) (17) Reducing the consumption of sugar, sweets and sweetened beverages (artificial juice, soda) (18) Changing eating habits: eating less at night, eating more at home and/or increasing the number of meals per day (19) Eating less fast food or junk food (20) Others: ________________ (77) Don't know (88) No if apply (99) No answ

V.10.2. And what was the maximum weight you reduced? ________ kg
(0) Didn't lose any weight (77) Don't know (88) No if apply (99) No answer

V.10.3. Was it with the support of a health professional?
(0) No (1) Yes (88) No if apply (99) No answer

V.10.3.1. From which professional? *Interviewer: check all to the options reported.*
(0) Doctor (1) Dietitian (2) Nurse (3) Professional of Education Physical (4) Others: ________ (77) Don't know (88) No if apply (99) No answer

V.11. In the last year, at the Health Center and/or City Gym, has any professional (doctor, nurse, dietitian, Physical Education professional, etc.) talked to you about obesity or recommended that you lose weight? *If not, skip to question V.12.*

(0) No (1) Yes (77) Don't know (99) No answer

V.11.1. Who normally introduced the conversation about obesity?

(0) Myself (1) The professional (77) Don't know (88) No if apply (99) No answer

V.12. What is the main difficulty that prevents you from losing weight? *Interviewer: check only one option.*

(0) Difficulty changing habits (1) Financial difficulties (2) Eating out (3) Lack of time to eat properly
(4) Lack of time to exercise (5) Lack of family support (6) Relapses (7) Other: _______________
(8) No difficulty (77) Don't know (99) No answer

VI) STIGMA

VI.1) Please indicate how much you agree with the item: Because of my body, I have suffered bullying or prejudice at various times in my life.

(1) I strongly disagree (2) I moderately disagree (3) I slightly disagree (4) I slightly agree
(5) I moderately agree (6) I strongly agree (99) No answer

VII.21. TEST " HOW IS YOUR DIET?" - I will ask you a few more questions about your diet. If you think more than one option is correct, choose the one you usually eat. *Interviewer: Use the form with the scale printed on it.*

VII.21.1. When I have small snacks throughout the day, I usually eat fruits or nuts.	(0) Never	(1) Rarely	(2) Lots of times	(3) Always
VII.21.2. When I choose fruits and vegetables, I give preference to those that are of locally produced.	(0) Never	(1) Rarely	(2) Lots of times	(3) Always
VII.21.3. When I choose fruits and vegetables, I prefer those that are organic.	(0) Never	(1) Rarely	(2) Lots of times	(3) Always
VII.21.4. I usually carry some food with me in case I feel hungry throughout the day.	(0) Never	(1) Rarely	(2) Lots of times	(3) Always
VII.21.5. I usually plan the meals I will eat during the day.	(0) Never	(1) Rarely	(2) Lots of times	(3) Always
VII.21.6. I usually vary my consumption of beans with peas, lentils or chickpeas.	(0) Never	(1) Rarely	(2) Lots of times	(3) Always
VII.21.7. In my house, we usually use whole wheat flour.	(0) Never	(1) Rarely	(2) Lots of times	(3) Always
VII.21.8. I usually eat fruit for breakfast.	(0) Never	(1) Rarely	(2) Lots of times	(3) Always
VII.21.9. I usually eat my meals sitting at the table.	(0) Never	(1) Rarely	(2) Lots of times	(3) Always
VII.21.10. I try to eat my meals calmly.	(0) Never	(1) Rarely	(2) Lots of times	(3) Always
VII.21.11. I usually participate in the preparation of food in my house.	(0) Never	(1) Rarely	(2) Lots of times	(3) Always
VII.21.12. In my house, we share the tasks that involve the preparation and consumption of meals.	(0) Never	(1) Rarely	(2) Lots of times	(3) Always
VII.21.13. I usually buy food at street markets or fairs.	(0) Never	(1) Rarely	(2) Lots of times	(3) Always
VII.21.14. I take advantage of meal times to take care of other things and end up not eating.	(3) Never	(2) Rarely	(1) Lots of times	(0) Always
VII.21.15. I usually eat my meals at my work desk.	(3) Never	(2) Rarely	(1) Lots of times	(0) Always
VII.21.16. I usually eat my meals sitting on the sofa in the living room or in bed.	(3) Never	(2) Rarely	(1) Lots of times	(0) Always
VII.21.17. I usually skip at least one of the main meals (lunch and/or dinner).	(3) Never	(2) Rarely	(1) Lots of times	(0) Always
VII.21.18. I usually eat candy, chocolate and other sweets.	(3) Never	(2) Rarely	(1) Lots of times	(0) Always

VII.21.19. I usually drink industrialized juices, such as juices from a carton, powder, bottle or can.	(3) Never	(2) Rarely	(1) Lots of times	(0) Always
VII.21.20. I usually go to fast food restaurants or snack bars.	(3) Never	(2) Rarely	(1) Lots of times	(0) Always
VII.21.21. I have the habit of "snacking" between meals.	(3) Never	(2) Rarely	(1) Lots of times	(0) Always
VII.21.22. I usually drink soda.	(3) Never	(2) Rarely	(1) Lots of times	(0) Always
VII.21.23. I usually swap lunch or dinner for sandwiches, savory snacks or pizza.	(3) Never	(2) Rarely	(1) Lots of times	(0) Always
VII.21.24. When I drink coffee or tea, I usually add sugar.	(3) Never	(2) Rarely	(1) Lots of times	(0) Always

VIII) OBSERVATIONS

____
____

VIII.1. End time: _____:_____

Interviewer, before releasing the user, check that all questions have been filled out correctly.

Figure S3. Reassesment questionnaire

UFMG

Qualificação do manejo da obesidade no SUS: efetividade de intervenção coletiva
Avaliação da efetividade de intervenção coletiva para o manejo da obesidade em usuários da Atenção Primária com indicação de cirurgia bariátrica

CUIDADO DA PESSOA COM OBESIDADE NO SUS

Data entry - Date: _____/_____/20____ Name: ____
Interviewer: Leave it to the field supervisor to fill in.

INDIVIDUAL ASSESSMENT INSTRUMENT

INTERVIEWER: PLEASE, FILL IN THE QUESTIONNAIRE IN PENCIL, WITH LETTER IN A LEGIBLE FORM.
COMPLETE QUESTIONS 1 AND 2 BEFORE STARTING THE INTERVIEW.

1. Interviewer: ____

2. Date of interview: _____/_____/20____ 3. Start time: _____:_____

4. Full Name:____

5. Number of identification: _______ *Interviewer: leave for the supervisor of field to fill in.*

6. Location: *Interviewer: no ask; just register the unit from the Academy from the City (PAC).*

(0) Diamante	(1) Parque das Águas	(2) Vale do Jatobá	(3) Regina
(4) Bairro Indústrias	(5) Petrópolis	(6) Milionários	(7) Vila Pinho
(8) Vila Santa Rita	(9) Lindéia	(10) Santa Lúcia	(11) Oswaldo Cruz
(12) Santa Rita Cássia	(13) Tia Amância	(14) Lab. Movimento	(15) Sagrada Família
(16) Paraíso	(17) São Geraldo	(18) Mariano de Abreu	(19) Boa Vista
(20) Riviera	(21) Jardim Belmonte	(22) Jardim Vitória	(23) São Marcos
(24) Dom Joaquim	(25) Ribeiro de Abreu	(26) União	(27) Santa Cruz
(28) Fazendinha	(29) Califórnia	(30) Ermelinda	(31) Mercado Lagoinha
(32) Centro Ref. Idoso	(33) CIAME	(34) Coqueiral	(35) Monte Azul
(36) Providência	(37) Via 240	(38) Zilah Spósito	(39) Campo Alegre
(40) Vila Biquinhas	(41) Jardim Felicidade	(42) Palmeiras (Uni BH)	(43) CRAS Vista Alegre
(44) Ventosa	(45) Conjunto Betânia	(46) Havaí	(47) Vila Leonina
(48) Confisco	(49) Jardim Alvorada	(50) São Francisco	(51) São José
(52) Serrano	(53) Santa Mônica	(54) Jardim Leblon	(55) Venda Nova
(56) Céu Azul	(57) Lagoa	(58) Serra Verde	

7. Are you attending the city gym activities? *Interviewer: if not, go to question 7.3.*
(0) No (1) Yes

7.1. If so, what day(s) do you work out at the city gym? *Interviewer: mark all the options reported.*
(0) Monday (1) Tuesday (2) Wednesday (3) Thursday (4) Friday (5) Saturday

7.2. If so, what time do you work out at the city gym?
(0) 07:00 (1) 08:00 (2) 09:00 (3) 10:00 (4) 11:00

7.3. What is the reason for not going to the city gym anymore
(0) Illness (1) Work/Study (2) Moving (3) Family care (4) Other: __________
(77) Don't know (88) No if apply (99) No answer

I) SOCIODEMOGRAPHIC PROFILE

I.1. Have you changed your address since___________ *(Interviewer: state the group's start date, consult the manual? Interviewr: if not, skip to question I.2.*
(0) No (1) Yes

I.1.1. If so, address: ____ I.2. Postal Code: ___________

I.2. Telephone: ( ) _____________________ I.3. Cell phone: ( ) ______________________________

I.4. What is the monthly family income? R$______________ (77) Don't know (88) No if apply
Minimum wage=R$ 1,212.00

I.5. How many people live in your home? ____________________

II) EATING HABITS

II.1. Normally, which meals you performs during the day: *Interviewer: ask each snack.*

II.1.1. Coffee from the morning?	(0) No	(1) Yes	(99) No answer
II.1.2. Snack from the morning?	(0) No	(1) Yes	(99) No answer
II.1.3. Lunch?	(0) No	(1) Yes	(99) No answer
II.1.4. Snack from the late?	(0) No	(1) Yes	(99) No answer
II.1.5. To have lunch or snack from the night?	(0) No	(1) Yes	(99) No answer
II.1.6. Supper or snack before of sleep?	(0) No	(1) Yes	(99) No answer

II.2. Number of meals in a typical day: _______ *Interviewer: do not ask; add up the answers above.*

Now I'm going to ask you some questions about your eating habits of certain foods. Always consider the average consumption of these foods.

II.3. In how many days from the week the Mr/Mrs. usually eat fruits.
______days Interviewer: *if response for five or more days a week, ask II.3.1*
(0) Never or less of one once a week (99) No answer

II.3.1. In general, how many times a day the Mr/Mrs. eat fruits?
(0) One time a day (1) Two times a day (2) Three times or more a day (88) No if apply

II.4. How many days a week do you usually eat at least one type of vegetable *(without count potatoes, cassava, taro or yam)* as lettuce, tomato, cabbage, carrot, chayote, eggplant, zucchini?
_______days *Interviewer: if response for five or more days a week, ask II.4.1.*
(0) Never or less of one once a week (99) No answer

II.4.1. In general, how many times a day the Mr/Mrs. usually eat that type of vegetable?
(0) One time a day (1) Two times a day (2) Three times or more a day (88) No if apply

II.5. In a day common, how many spoons (soup) of vegetables do you eat?
______spoons/day (88) No if apply (99) No answer

II.5.1. Mode of preparation: (0) Raw (1) Sautéed/Cooked (88) No if apply (99) No asnwer

II.6. In a day common, how many spoons (soup) of vegetables do you eat?
______spoons/day (88) No if apply (99) No answer

II.6.1. Mode of preparation: (0) Raw (1) Sautéed/Cooked (88) No if apply (99) No asnwer

II.7. In how many days from the week the Mr/Mrs. usually eat bean?
______days (0) Never or less of one once a week (99) No answer

II.8. In how many days from the week, the Mr/Mrs. usually eat meat red (ox, pork, kid, goat, sheep, etc.)?
______days (0) Never or less of one once a week (99) No answer

II.9. In how many days from the week the Mr/Mrs. usually eat chicken/hen?
______days (0) Never or less of one once a week (99) No answer

II.10. In how many days from the week the Mr/Mrs. usually eat fish?
______days (0) Never or less of one once a week (99) No answer

II.11. In how many days from the week the Mr/Mrs. usually take juice of box/tin or soft drink in dust?
______days (0) Never or less of one once a week (99) No answer

II.12. In how many days from the week the Mr/Mrs. usually take refrigerator?
______days (0) Never or less of one once a week (99) No answer

II.13. In how many days from the week the Mr/Mrs. usually take milk? (of animal origin: cow, goat, buffalo, etc.)
______days (0) Never or less of one once a week (99) No answer

II.14. In how many days from the week the Mr/Mrs. usually eat food candy as stuffed biscuits/cookies, chocolate, jelly, candy and others?
______days (0) Never or less of one once a week (99) No answer

II.15. In how many days from the week the Mr/Mrs. usually eat drink chocolate milk or yogurt with flavor?
______days (0) Never or less of one once a week (99) No answer

II.16. In how many days from the week the Mr/Mrs. usually eat food as sausage, sausage, mortadella or ham?
______days (0) Never or less of one once a week (99) No answer

II.17. In how many days from the week the Mr/Mrs. usually eat food as bread of form, hot dog or hamburger?
______days (0) Never or less of one once a week (99) No answer

II.18. How many days last 1 kg of salt in the your home? ______days (99) No answer

II.18.1. How many people live in your household? __________
Interviewer: leave to the supervisor field to fill in. Supervisor: consult the Screening questionnaire.

II.19. How much sugar do you use in one month? _______ kg (99) No answer
Interviewer: If don't buy sugar, put zero.

II.19.1. How many people live in your household? __________
Interviewer: leave to the supervisor field to fill in. Supervisor: consult the Screening questionnaire.

II.20. What type of fat and used, *with bigger frequency*, in your home to sauté, fry or to roast the food?
Interviewer: Please check only one option.
(0) Oil olive (1) Oil vegetable (2) Butter (3) Margarine/cream/shortening vegetable (4) Lard/fat animal
(5) No use fat (6) Others: ___________ (99) No answer

II.20.1. Amount monthly from the fat reported: __________ *Interviewer: remember what one bottle of oil he has 900mL*
(88) No if apply (99) No asnwer

II.20.2. Number of people who consume fat: _________ *Interviewer: Pay attention to whether there are people who only have one meal at home and the possible differences in the number of people who eat at home during the weekend.*
(88) No if apply (99) No asnwer

III) REASONS FOR FOOD CHOICES. *Interviewer: Use the form with the scale printed on it.*

I eat what I eat, ...

		**III.1. Preference**			
III.1.1 ... because I have willing of eat.	(1) Never	(2) Rarely	(3) To the times	(4) Often	(5) Always
III.1.2 ... because is yummy.	(1) Never	(2) Rarely	(3) To the times	(4) Often	(5) Always
III.1.3 ... because I like it.	(1) Never	(2) Rarely	(3) To the times	(4) Often	(5) Always
		**III.2. Habits**			
III.1.1 ... because I am accustomed the eat that.	(1) Never	(2) Rarely	(3) To the times	(4) Often	(5) Always
III.1.2 ... because is what I generally eat.	(1) Never	(2) Rarely	(3) To the times	(4) Often	(5) Always
III.1.3. ... because I know the product.	(1) Never	(2) Rarely	(3) To the times	(4) Often	(5) Always
		**III.3. Need and hunger**			
III.3.1. ... because I need energy.	(1) Never	(2) Rarely	(3) To the times	(4) Often	(5) Always
III.3.2. ... because it satisfies my hunger in a pleasant way.	(1) Never	(2) Rarely	(3) To the times	(4) Often	(5) Always
III.3.3. ...because I have hunger.	(1) Never	(2) Rarely	(3) To the times	(4) Often	(5) Always
		**III.4. Health**			
III.4.1. ... for to maintain food balanced.	(1) Never	(2) Rarely	(3) To the times	(4) Often	(5) Always
III.4.2. ... because is healthy.	(1) Never	(2) Rarely	(3) To the times	(4) Often	(5) Always
III.4.3. ... because it keeps me energized and motivated.	(1) Never	(2) Rarely	(3) To the times	(4) Often	(5) Always
		**III.5. Convenience**			
III.5.1. ... because is fast to prepare.	(1) Never	(2) Rarely	(3) To the times	(4) Often	(5) Always
III.5.2. ... because is convenient.	(1) Never	(2) Rarely	(3) To the times	(4) Often	(5) Always

III.5.3. ... because is easy to prepare.	(1) Never	(2) Rarely	(3) To the times	(4) Often	(5) Always
**III.6. Pleasure**					
III.6.1. ... because it gives me pleasure.	(1) Never	(2) Rarely	(3) To the times	(4) Often	(5) Always
III.6.2. ... for me give something really special.	(1) Never	(2) Rarely	(3) To the times	(4) Often	(5) Always
III.6.3. ... to me reward.	(1) Never	(2) Rarely	(3) To the times	(4) Often	(5) Always
**III.7. Traditional food**					
III.7.1. ... because belongs the right situations.	(1) Never	(2) Rarely	(3) To the times	(4) Often	(5) Always
III.7.2. ... by tradition (ex.: tradition of family, special occasions)	(1) Never	(2) Rarely	(3) To the times	(4) Often	(5) Always
III.7.3. ... because I grew up eating like this.	(1) Never	(2) Rarely	(3) To the times	(4) Often	(5) Always
**III.8. Natural questions**					
III.8.1. ... because is natural.	(1) Never	(2) Rarely	(3) To the times	(4) Often	(5) Always
III.8.2. ... because no contains harmful substances (e.g. pesticides, pollutants, antibiotics).	(1) Never	(2) Rarely	(3) To the times	(4) Often	(5) Always
III.8.3. ... because is organic.	(1) Never	(2) Rarely	(3) To the times	(4) Often	(5) Always
**III.9. Socialization**					
III.9.1. ... because it is part of a social situation.	(1) Never	(2) Rarely	(3) To the times	(4) Often	(5) Always
III.9.2. ... to what I may to spend time with other people.	(1) Never	(2) Rarely	(3) To the times	(4) Often	(5) Always
III.9.3. ... because social meetings become more enjoyable.	(1) Never	(2) Rarely	(3) To the times	(4) Often	(5) Always
**III.10. Price**					
III.10.1. ... because is cheap.	(1) Never	(2) Rarely	(3) To the times	(4) Often	(5) Always
III.10.2. ... because I don't want to spend a lot of money.	(1) Never	(2) Rarely	(3) To the times	(4) Often	(5) Always
III.10.3. ... because it is in promotion.	(1) Never	(2) Rarely	(3) To the times	(4) Often	(5) Always
**III.11. Visual attraction**					
III.11.1. ... because the presentation is attractive (e.g. packaging).	(1) Never	(2) Rarely	(3) To the times	(4) Often	(5) Always
III.11.2. ... because it immediately catches my attention (presentation in the supermarket, it's colorful).	(1) Never	(2) Rarely	(3) To the times	(4) Often	(5) Always
III.11.3. ... because I recognize from advertisements or I've seen it on TV.	(1) Never	(2) Rarely	(3) To the times	(4) Often	(5) Always
**III.12. Weight control**					
III.12.1. ... because has few calories.	(1) Never	(2) Rarely	(3) To the times	(4) Often	(5) Always
III.12.2. ... because I control my weight.	(1) Never	(2) Rarely	(3) To the times	(4) Often	(5) Always
III.12.3. ... because has little fat.	(1) Never	(2) Rarely	(3) To the times	(4) Often	(5) Always
**III.13. Emotion control**					
III.13.1. ... because I am sad.	(1) Never	(2) Rarely	(3) To the times	(4) Often	(5) Always
III.13.2. ... because I am frustrated.	(1) Never	(2) Rarely	(3) To the times	(4) Often	(5) Always
III.13.3. ... because I feel alone.	(1) Never	(2) Rarely	(3) To the times	(4) Often	(5) Always

III.14. Social standards					
III.14.1. ... because it would be impolite not to eat.	(1) Never	(2) Rarely	(3) To the times	(4) Often	(5) Always
III.14.2. ... for avoid disappoint somebody what is trying to please me.	(1) Never	(2) Rarely	(3) To the times	(4) Often	(5) Always
III.14.3. ... because I have to eat.	(1) Never	(2) Rarely	(3) To the times	(4) Often	(5) Always
**III.15. Social image**					
III.15.1. ... because it's in fashion.	(1) Never	(2) Rarely	(3) To the times	(4) Often	(5) Always
III.15.2. ... because it makes me project a good image to others.	(1) Never	(2) Rarely	(3) To the times	(4) Often	(5) Always
III.15.3. ... because others like it.	(1) Never	(2) Rarely	(3) To the times	(4) Often	(5) Always

IV) TRUST TO ABILITY CULINARY – Index of Skills Culinary (ISC). *Interviewer: Use the record with the scale printed.*

IV.1. How trusting you if feel in:

IV.1.1. Sauté a food	(0) Not confident at all	(1) Not very confident	(2) Trusting	(3) Very confident
IV.1.2. Bake in the oven	(0) Not confident at all	(1) Not very confident	(2) Trusting	(3) Very confident
IV.1.3. Season meat using only natural seasonings	(0) Not confident at all	(1) Not very confident	(2) Trusting	(3) Very confident
IV.1.4. Follow a simple recipe	(0) Not confident at all	(1) Not very confident	(2) Trusting	(3) Very confident
IV.1.5. Make a homemade tomato sauce, only with tomatoes and natural ingredients	(0) Not confident at all	(1) Not very confident	(2) Trusting	(3) Very confident
IV.1.6. Prepare a homemade soup	(0) Not confident at all	(1) Not very confident	(2) Trusting	(3) Very confident
IV.1.7. Cooking beans in a pressure cooker	(0) Not confident at all	(1) Not very confident	(2) Trusting	(3) Very confident
IV.1.8. Grilling meat	(0) Not confident at all	(1) Not very confident	(2) Trusting	(3) Very confident
IV.1.9. Prepare a simple homemade cake	(0) Not confident at all	(1) Not very confident	(2) Trusting	(3) Very confident
IV.1.10. Preparing lunch or dinner by combining foods and ingredients that are already in the house and without needing a recipe	(0) Not confident at all	(1) Not very confident	(2) Trusting	(3) Very confident

V) HEALTH PROFILE – Part 1

V.1. In general, as you evaluates the your health? *Interviewer: read to the alternatives.*
(1) Very bad (2) Bad (3) Regular (4) Good (5) Very good (99) No answer

V.2. How many days a week you usually to practice exercise physical or sport (e.g.: walking, running, weight training, water aerobics, stretching, pilates, team games, dancing, etc.) ? __________ days (99) No answer

V.2.1. On the day you do physical exercise or sport, how long does this activity last?
________ minutes (88) No if apply

V.3. Would you like to weigh... *Interviewer: read the alternatives.*
(1) More (2) Less (3) Equal (77) Don't know (99) No answer

V.4. Have you ever tried to lose weight? *Interviewer: If no, jump to question V.5.*
(0) No (1) Yes (77) Don't know (99) No answer

V.4.1. If so, how long? _______ years ________ months (88) No if apply

V.4.2. If so, how did you try to lose weight? *Interviewer: check all the reported options*
(0) Eating less food (1) Eating less caloric food (2) Eating less fat (3) Eating less carbohydrates

(4) Exercising (5) Skipping meals/fasting (6) Using diet products (7) Using a liquid diet (8) With a weight loss program (9) Following a special diet (Dr. Atkins, lowcarb...) (10) With medication prescribed by a doctor (11) With over-the-countermedications, herbs or supplements (12) Starting or returning to smoking (13) With laxatives or vomiting (14) Having surgery, such as gastric Bypass (15) Drinking lots of water (16) Eating more fruits, vegetables (salads) (17) Reducing the consumption of sugar, sweets and sweetened beverages (artificial juice, soda) (18) Changing eating habits: eating less at night, eating more at home and/or increasing the number of meals per day (19) Eating less fast food or junk food (20) Others: ________________ (77) Don't know (88) No if apply (99) No answ

V.4.3. Was it with the support of a health professional?
(0) No (1) Yes (88) No if apply (99) No answer

V.4.3.1. From which professional? *Interviewer: check all to the options reported.*
(0) Doctor (1) Dietitian (2) Nurse (3) Professional of Education Physical (4) Others: ________ (77) Don't know (88) No if apply (99) No answer

V.5. How often do you weigh yourself?
(0) Every day (including saturday and sunday) (1) 2-3 days per week (2) 1 day per week (3) Once every 2 weeks (4) Once a month (5) Once every 2 months (6) A few times a year (7) Never (77) Don't know (99) No answer

Part 2: Indication for bariatric surgery

V.6. Are you currently awaiting bariatric surgery?
(0) No (1) Yes (77) Don't know (88) No if apply (99) No answer

V.7. In the last six months, has a doctor ever diagnosed you with...

V.7.1. Cardiovascular disease (ex.: coronary artery disease, coronary heart disease, congestive heart failure, stroke)?
(0) No (1) Yes (77) Don't know (88) No if apply (99) No answer

V.7.2. High cholesterol and/or triglycerides?
(0) No (1) Yes (77) Don't know (88) No if apply (99) No answer

V.7.3. Sleep apnea (pauses in breathing while sleeping)?
(0) No (1) Yes (77) Don't know (88) No if apply (99) No answer

V.7.4. Joint disease/Osteoarthritis?
(0) No (1) Yes (77) Don't know (88) No if apply (99) No answer

V.7.5. Diabetes?
(0) No (1) Yes (77) Don't know (88) No if apply (99) No answer

V.7.6. High blood pressure?
(0) No (1) Yes (77) Don't know (88) No if apply (99) No answer

V.8. Do you currently smoke any tobacco products? *Interviewer: consider the following examples: cigarettes, straw cigarettes, clove or Bali cigarettes, pipes, cigarillos, hookah, etc.*
(0) Not currently smoke (1) Yes, less than daily (2) Yes, daily (88) No if apply (99) No answer

VI) STIGMA

VI.1) Please indicate how much you agree with the item: Because of my body, I have suffered bullying or prejudice at various times in my life.
(1) I strongly disagree (2) I moderately disagree (3) I slightly disagree (4) I slightly agree
(5) I moderately agree (6) I strongly agree (99) No answer

VII) STAGE OF CHANGE, SELF-EFFICACY AND DECISION BALANCE FOR WEIGHT REDUCTION

VII.1. Identifying the stage of change to reduce weight. *Interviewer: ask the relevant questions, and at the end check ONLY ONE STAGE.*

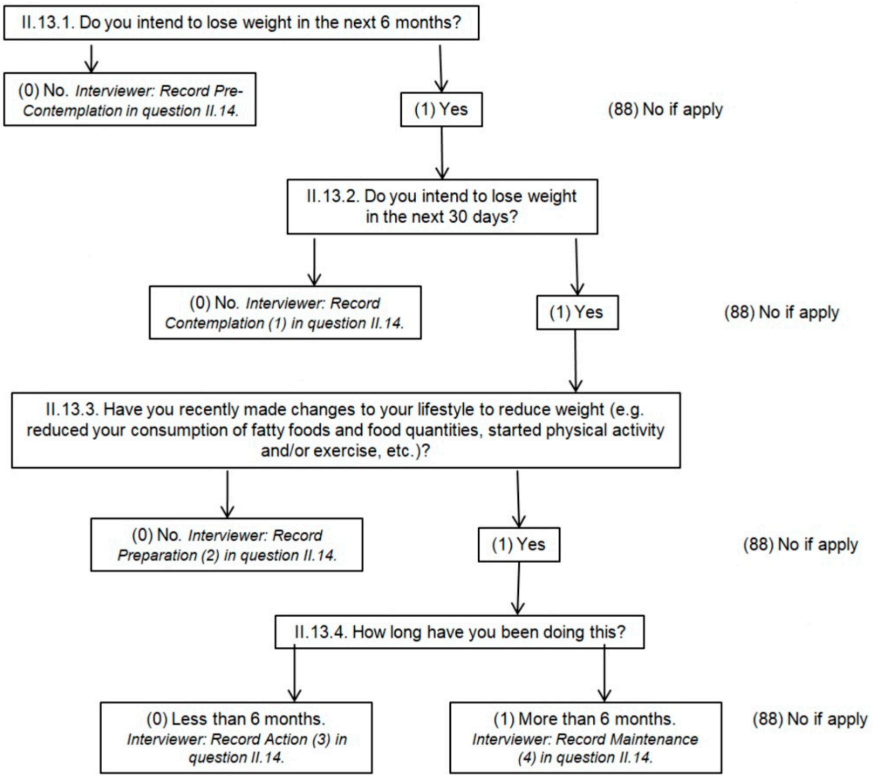

VII.2. Result: *Interviewer: don't ask this question; just check one option.*

(0) Pre-Contemplation (1) Contemplation (2) Preparation (3) Action (4) Maintenance (88) No if apply

VII.3. Identification of self-efficacy to reduce weight.

How confident are you that you can lose weight even if... *Interviewer: read the answer options for each situation. Use the form with the scale printed on it.*

VII.3.1. ...need a lot of time to improve your practices?	(1) Not at all confident	(2) Somewhat confident	(3) Moderately confident	(4) Very confident	(5) Completely confident	(88) No if apply
VII.3.2. ...need many attempts until you succeed?	(1) Not at all confident	(2) Somewhat confident	(3) Moderately confident	(4) Very confident	(5) Completely confident	(88) No if apply
VII.3.3. ...need to rethink your strategies?	(1) Not at all confident	(2) Somewhat confident	(3) Moderately confident	(4) Very confident	(5) Completely confident	(88) No if apply

VII.4. Result. *Interviewer: don't ask this question; just check one option.*

(0) Low self-efficacy (1) High self-efficacy **(At least 2 answers (4) or (5) in II.15)** (88) No if apply

VII.5. Identifying the balance of decisions to reduce weight.

Indicate how important each of the situations below is to you at the moment. *Interviewer: read the answer options for each situation. Use the form with the printed scale.*

VII.5.1. Plan more carefully when buying and/or preparing food to contribute to weight loss.	(1) Not at all important	(2) Slightly important	(3) Important	(4) Very important	(88) No if apply	(99) No answer

VII.5.2. Feel good about taking care of your health during the weight loss process.	(1) Not at all important	(2) Slightly important	(3) Important	(4) Very important	(88) No if apply	(99) No answer
VII.5.3. Learn to deal with frustrations that arise during the weight loss process.	(1) Not at all important	(2) Slightly important	(3) Important	(4) Very important	(88) No if apply	(99) No answer
VII.5.4. Be able to identify the situations in your daily life that hinder weight loss.	(1) Not at all important	(2) Slightly important	(3) Important	(4) Very important	(88) No if apply	(99) No answer
**Indicate how worrying each of the situations below is for you at the moment.** *Interviewer: Read the response options for each situation. Use the form with the printed scale.*						
VII.5.5. Returning to some behavior that hinders weight loss.	(1) Not at all worrying	(2) Slightly worrying	(3) Worrying	(4) Very worrying	(88) No if apply	(99) No answer
VII.5.6. Feeling unsupported in the weight loss process.	(1) Not at all worrying	(2) Slightly worrying	(3) Worrying	(4) Very worrying	(88) No if apply	(99) No answer
VII.5.7. Having little time available to include in your daily routine the changes necessary for weight loss.	(1) Not at all worrying	(2) Slightly worrying	(3) Worrying	(4) Very worrying	(88) No if apply	(99) No answer
VII.5.8. Being discouraged by family and/or friends when starting a new attempt to lose weight.	(1) Not at all worrying	(2) Slightly worrying	(3) Worrying	(4) Very worrying	(88) No if apply	(99) No answer

VIII) PARTICIPATION IN WEIGHT LOSS INTERVENTIONS

Which group did the user participate in? *Interviewer: don't ask, just record.*
(0) Intervention group (1) Control group

Part 1 - ONLY users participating in the CONTROL GROUP

VIII.1. In the last six months, have you participated in any activities with guidance on healthy eating? *Interviewer: if so, ask VIII.1.1.*
(0) No (1) Yes (77) Don't know (88) No if apply (99) No answer

VIII.1.1. If so, what type of activity was it? *Interviewer: check all that apply.*
(0) Group activity at the health center (e.g., group, waiting room, etc.) (1) Group activity elsewhere
(2) Health center consultation (3) Specialty center consultation (4) Private consultation
(5) Home visit by health center staff (6) Other activity(ies): __________ (77) Don't know (88) No if apply
(99) No answer

Part 2 - ONLY users participating in the INTERVENTION GROUP

Now let's talk about the activities of the obesity treatment group that you participated in here at Academia da Cidade. ***Interviewer: If the user does not remember, provide the necessary additional information.***

VIII.2. Have you felt any benefit since you concluded your participation in the group?
(0) No (1) Yes (77) Don't know (88) No if apply (99) No answer

VIII.2.1. If so, what were the benefit(s):
(1) Weight loss (2) Greater energy (3) Improvement in laboratory tests
(4) Greater knowledge about food and nutrition, facilitating healthier food choices (5) Improved health
(6) Others: __________________ (77) Don't know (88) No if apply (99) No answer

VIII.3. Have you missed any group meetings? *Interviewer: if not, skip to question VIII.4.*
(0) No (1) Yes (77) Don't know (88) No if apply (99) No answer

VIII.3.1. If so, what was the main reason for your absence? *Interviewer: read the options and mark only the main difficulty presented by the user.*
(0) Lack of willpower/motivation (1) Lack of social support (support from family, friends) (2) Lack of time
(3) Health problem (4) Health problem of family member (5) Other: _______________

(77) Don't know (88) No if apply (99) No answer

VIII.4. What was the biggest difficulty you faced in putting into practice the guidelines shared in the group? *Interviewer: read the options and check only the option that corresponds to the main difficulty presented by the user.*
(0) Lack of money (1) Lack of motivation (2) Lack of support (e.g.: family, friends) (3) Lack of knowledge
(4) Lack of time (5) Health problem (6) Lack of professional support (7) No difficulty
(8) Other: ________________ (77) Don't know (88) No if apply (99) No answer

VIII.5. How satisfied are you with the team that ran the group meetings? *Interviewer: read the options.*
(0) Very dissatisfied (1) Dissatisfied (2) Neither satisfied nor dissatisfied (3) Satisfied (4) Very satisfied
(77) Don't know (88) No if apply (99) No answer

VIII.6. What would you change about the obesity group that you participated in here at the city gym? *Interviewer: Read all the options below.*
(0) Team that carried out the activities (1) Meeting location (2) Meeting dates (3) Meeting times
(4) Duration of each meeting (60 min) (5) Total number of meetings (TG1: 7; TG2: 9) (6) Total duration of the group (6 months) (7) Time interval between meetings (15 to 45 days) (8) Topics of one or more meetings
(9) One or more ICT activities (message/phone call or postcard) (10) Other: _________ (11) Not change anything
(77) Don't know (88) No if apply (99) No answer

VIII.7. Would you recommend the group to a relative, friend, neighbor or acquaintance to participate in?
(0) No (1) Yes (77) Don't know (88) No if apply (99) No answer

VIII.8. During the group activities, were your doubts clarified?
(0) No (1) Partially (2) Yes (3) No doubts (77) Don't know (88) No if apply (99) No answer

VIII.9. Did you miss any topic being discussed during the group meetings?
(0) No (1) Yes (77) Don't know (88) No if apply (99) No answer

VIII.10. After ______ (*Interviewer: state the group's start date; consult the manual*), did you participate in other weight loss activities? *(Consider any activities other than the research intervention)*
(0) No (1) Yes (77) Don't know (88) No if apply (99) No answer

VIII.10.1. If so, what type of activity was it? *Interviewer: check all that apply.*
(0) Group activity at the health center (e.g., group, waiting room, etc.) (1) Group activity elsewhere
(2) Health center consultation (3) Specialty center consultation (4) Private consultation
(5) Home visit by health center staff (6) Other activity(ies): __________ (77) Don't know (88) No if apply
(99) No answer

IX) ANTHROPOMETRY WITH ALL USERS

IX.1. Waist Circumference (WC):
1st measure: _____cm 2nd measure: _____cm 3rd measure: _____cm Average: _____cm

IX.2. Weight: __________ kg (99) No answer

IX.3. Baseline weight: __________ kg *Interviewer: check list with available baseline data.* (99) No answer

IX.4. % weight reduction: ___________ *Interviewer: do not ask; calculate % weight reduction.* (88) No if apply

IX.4.1. Which group did the interviewee participate in? *Interviewer: do not ask; just record.*
(0) TG1 (1) TG2

IX.4.1.1. If TG1, is the % weight reduction less than 3%? *Interviewer: do not ask; just record.*
(0) No (1) Yes *Interviewer: ask IX.4.2.* (88) No if apply

IX.4.1.2. If GT2, is the % weight reduction less than 5%? *Interviewer: do not ask; just record*
(0) No (1) Yes *Interviewer: ask IX.4.2.* (88) No if apply

IX.4.2. What was the main difficulty that prevented you from losing weight/reaching the goal proposed by the group?
(0) Difficulty changing habits (1) Financial difficulties (2) Eating meals outside the home
(3) Lack of time to eat properly (4) Lack of time to exercise (5) Lack of family support
(6) Relapses (7) Other: ____________ (8) No difficulty
(77) Don't know (88) No if apply (99) No answer

**X. TEST " HOW IS YOUR DIET?" - I will ask you a few more questions about your diet. If you think more than one option is correct, choose the one you usually eat.** *Interviewer: Use the form with the scale printed on it.*				
X.1. When I have small snacks throughout the day, I usually eat fruits or nuts.	(0) Never	(1) Rarely	(2) Lots of times	(3) Always
X.2. When I choose fruits and vegetables, I give preference to those that are of locally produced.	(0) Never	(1) Rarely	(2) Lots of times	(3) Always
X.3. When I choose fruits and vegetables, I prefer those that are organic.	(0) Never	(1) Rarely	(2) Lots of times	(3) Always
X.4. I usually carry some food with me in case I feel hungry throughout the day.	(0) Never	(1) Rarely	(2) Lots of times	(3) Always
X.5. I usually plan the meals I will eat during the day.	(0) Never	(1) Rarely	(2) Lots of times	(3) Always
X.6. I usually vary my consumption of beans with peas, lentils or chickpeas.	(0) Never	(1) Rarely	(2) Lots of times	(3) Always
X.7. In my house, we usually use whole wheat flour.	(0) Never	(1) Rarely	(2) Lots of times	(3) Always
X.8. I usually eat fruit for breakfast.	(0) Never	(1) Rarely	(2) Lots of times	(3) Always
X.9. I usually eat my meals sitting at the table.	(0) Never	(1) Rarely	(2) Lots of times	(3) Always
X.10. I try to eat my meals calmly.	(0) Never	(1) Rarely	(2) Lots of times	(3) Always
X.11. I usually participate in the preparation of food in my house.	(0) Never	(1) Rarely	(2) Lots of times	(3) Always
X.12. In my house, we share the tasks that involve the preparation and consumption of meals.	(0) Never	(1) Rarely	(2) Lots of times	(3) Always
X.13. I usually buy food at street markets or fairs.	(0) Never	(1) Rarely	(2) Lots of times	(3) Always
X.14. I take advantage of meal times to take care of other things and end up not eating.	(3) Never	(2) Rarely	(1) Lots of times	(0) Always
X.15. I usually eat my meals at my work desk.	(3) Never	(2) Rarely	(1) Lots of times	(0) Always
X.16. I usually eat my meals sitting on the sofa in the living room or in bed.	(3) Never	(2) Rarely	(1) Lots of times	(0) Always
X.17. I usually skip at least one of the main meals (lunch and/or dinner).	(3) Never	(2) Rarely	(1) Lots of times	(0) Always
X.18. I usually eat candy, chocolate and other sweets.	(3) Never	(2) Rarely	(1) Lots of times	(0) Always
X.19. I usually drink industrialized juices, such as juices from a carton, powder, bottle or can.	(3) Never	(2) Rarely	(1) Lots of times	(0) Always
X.20. I usually go to fast food restaurants or snack bars.	(3) Never	(2) Rarely	(1) Lots of times	(0) Always
X.21. I have the habit of “snacking” between meals.	(3) Never	(2) Rarely	(1) Lots of times	(0) Always
X.22. I usually drink soda.	(3) Never	(2) Rarely	(1) Lots of times	(0) Always
X.23. I usually swap lunch or dinner for sandwiches, savory snacks or pizza.	(3) Never	(2) Rarely	(1) Lots of times	(0) Always
X.24. When I drink coffee or tea, I usually add sugar.	(3) Never	(2) Rarely	(1) Lots of times	(0) Always

XI) OBSERVATIONS

____

____

XI.1. End time: _____:_____

Interviewer, before releasing the user, check that all questions have been filled out correctly.

Figure S4. Description of the ICT activities.

Postcard: Your presence matters

FRONT

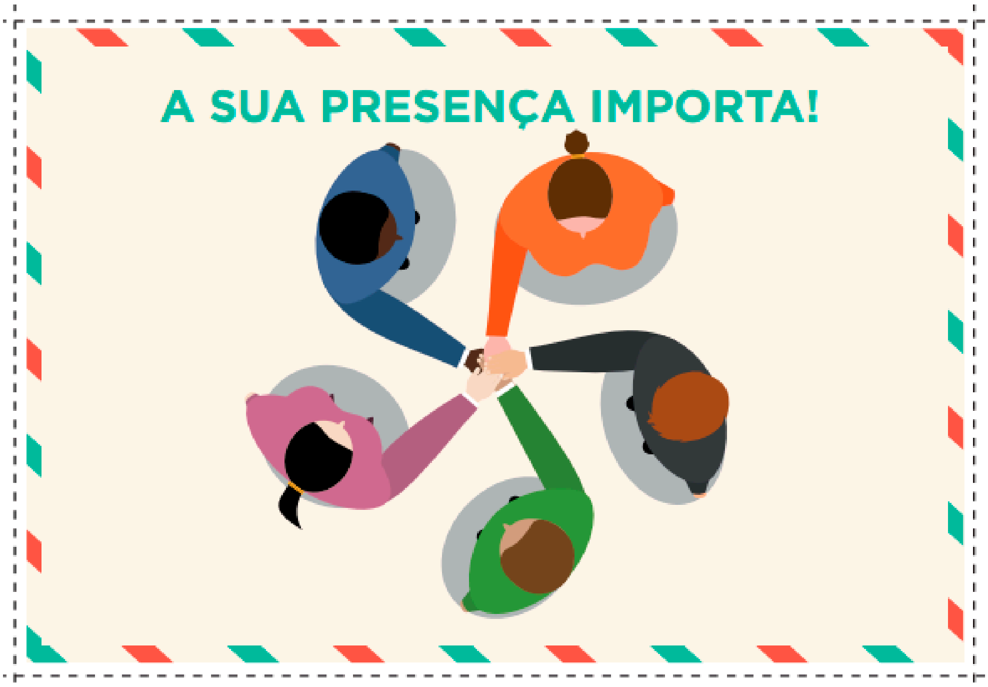

BACK

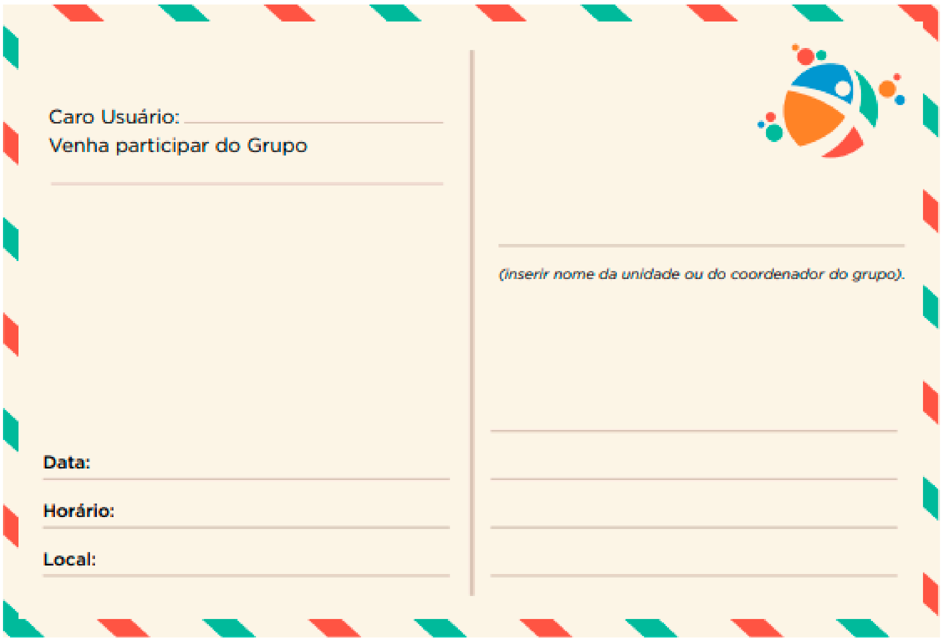

Postcard: Gains and challenges along the way

FRONT

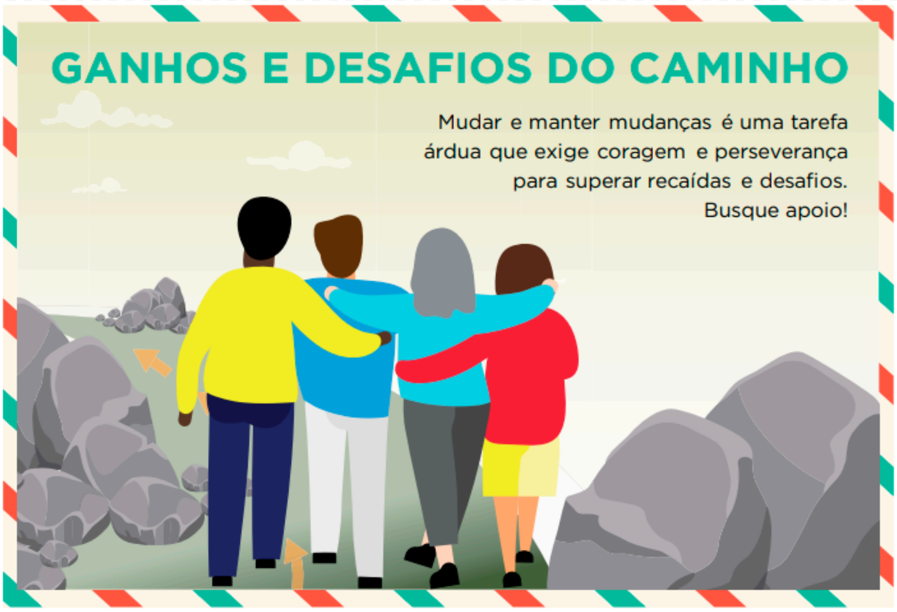

BACK

Caro Usuário: ________________
Venha participar do Grupo ________________

Data: ________________

Horário: ________________

Local: ________________

____

(inserir nome da unidade ou do coordenador do grupo).

Obrigado(a) pela sua presença nos encontros. É muito importante estarmos e permanecermos juntos.

Todo caminho tem obstáculos; porém, quando caminhamos juntos somos mais fortes! Conte conosco!

Postcard: Importance of changing

FRONT

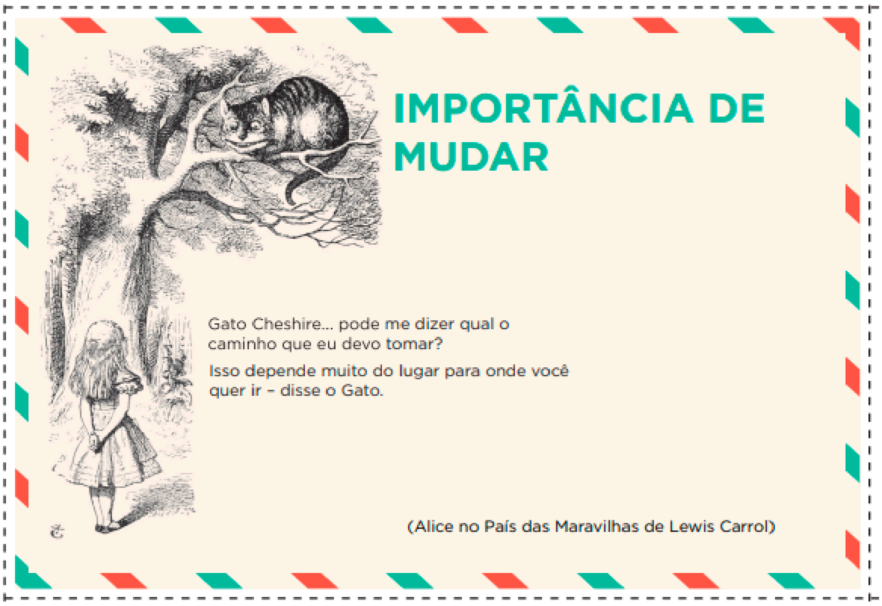

BACK

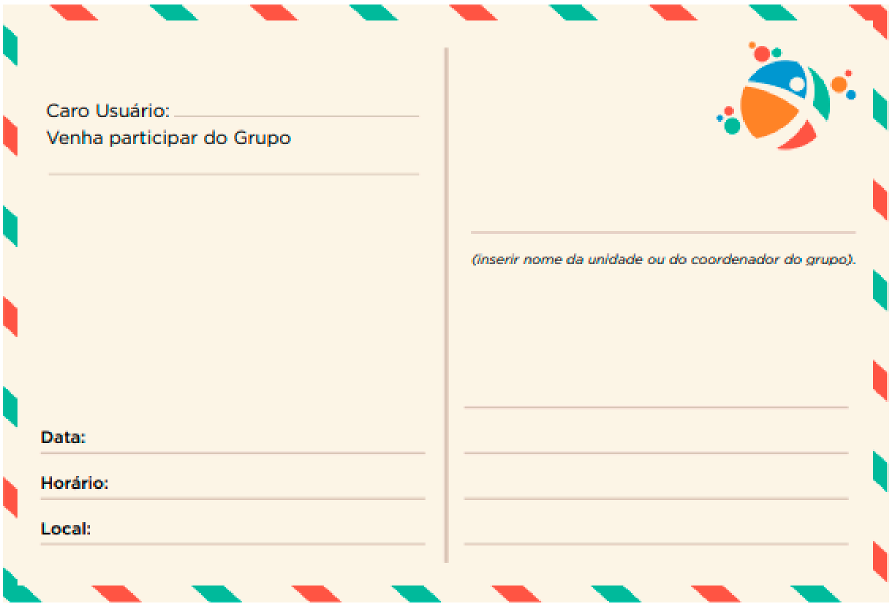

Postcard: Take care of yourself today and everyday

FRONT

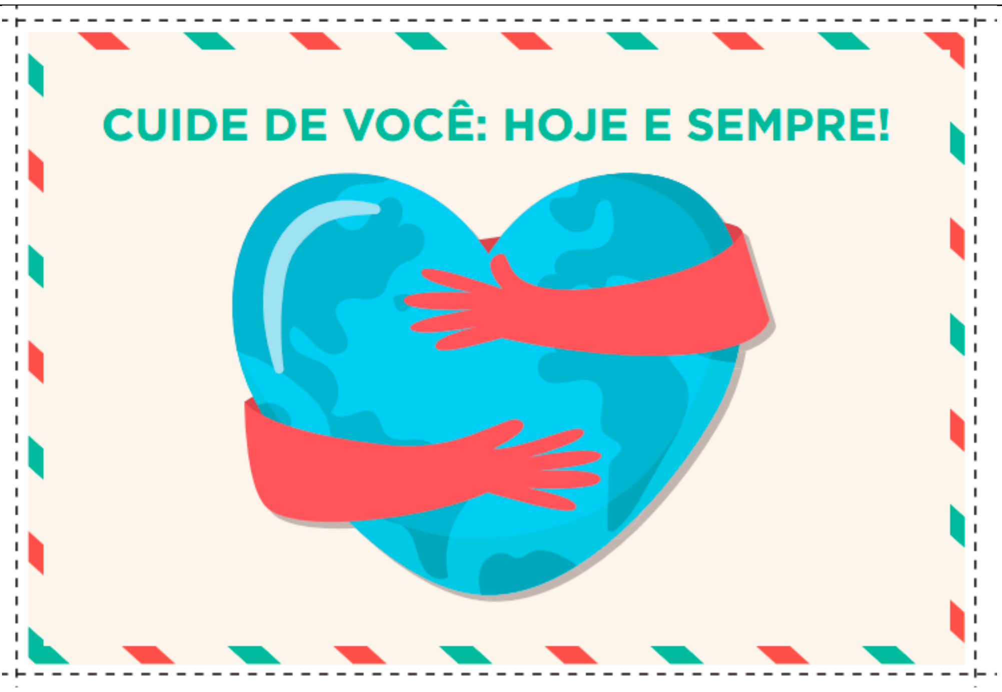

BACK

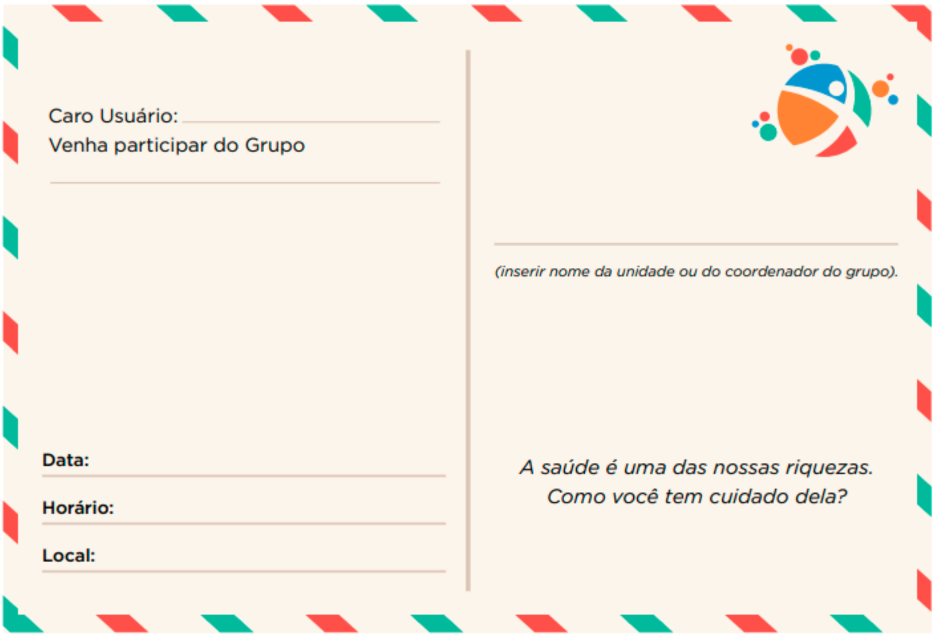

Postcard: Health + flavor

FRONT

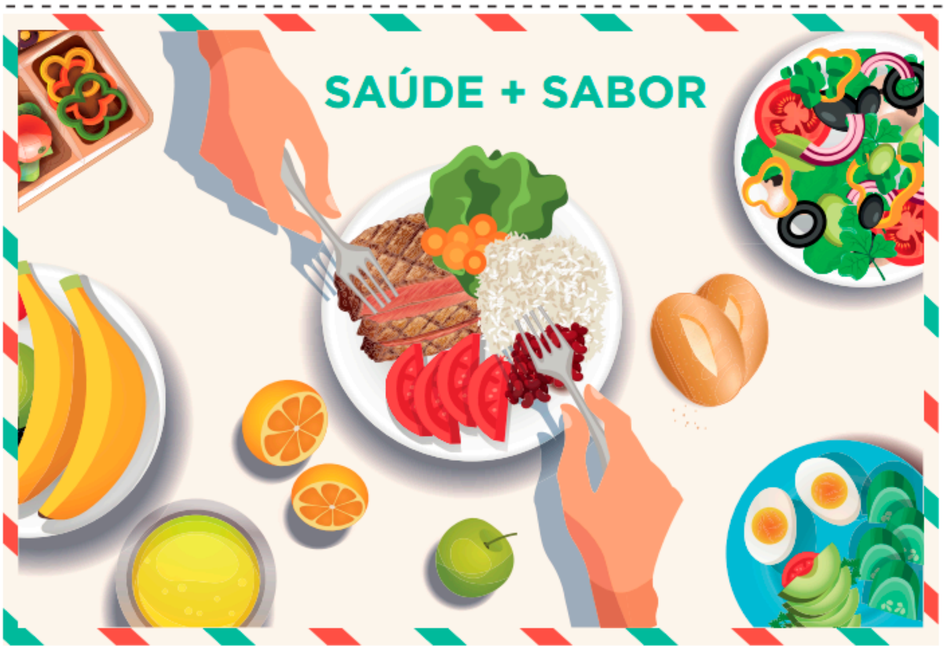

BACK

Caro Usuário: ______

Venha participar do Grupo

______

______

(inserir nome da unidade ou do coordenador do grupo).

Data:

Horário:

Local:

A alimentação saudável que contribui para reduzir peso não deve ser sinônimo de sofrimento ou falta de sabor.

Comida de verdade pode e deve ser rica em cores, sabores e texturas. Teste novas formas de preparar, servir e combinar os alimentos!

**Message or Phone Call:** Our Goals		
**Message via app**	**Call to those who attended the face-to-face meeting**	**Call to those who did not attend the face-to-face meeting**
Thank you for participating in our last meeting. Remember that planning is important to be successful. Think about the goals you have set. We look forward to seeing you at the next meeting (date, place and time).	*Hello, my name is... (identify yourself), I am part of the team at the health unit where you participate in the Group... (group name). Thank you for participating in the last meeting. Remember: to be successful it is important to plan. Think about the goals you have set. See you at the next meeting (date, place and time)!*	*Hello, my name is... (identify yourself), I am part of the at the health unit where you participate in the Group... (group name). You were missed at the last meeting! I would like to remind you that in order to change, you need to plan and create goals. Until the next meeting (date, place and time)!*

**Message or phone call:** You are important		
**Message via app**	**Call to those who attended the face-to-face meeting**	**Call to those who did not attend the face-to-face meeting**
Thank you for participating in the Group... (group name). Your participation is important and the topics covered are of interest to you. If you have any questions, please contact	Hello, my name is... (identify yourself), I am a member of the team at the health unit where you participate in the Group... (group name). I called to thank you for participating in the group and to remind	Hello, my name is... (identify yourself), I am part of the team at the health unit where you participate in the Group... (say the name of the group). We missed you! See you at the next

the health unit. Until the next meeting (date, place and time).	you how important you are. Until the next meeting (date, place and time)!	meeting (date, place and time)!

Message or phone call: Action plan underway

Message via app	Call to those who attended the face-to-face meeting	Call to those who did not attend the face-to-face meeting
Planning can help you achieve your goals, overcoming obstacles and defining strategies for change! Remember the Action Plan we prepared. We look forward to seeing you at the next meeting (date, place and time).	*Hello, my name is... (identify yourself), I am part of the team at the health unit where you participate in the Group... (name of the group). I called to remind you that the planning we do together can help you achieve your goals and overcome obstacles! Thank you for participating. See you at the next meeting (date, place and time)!*	*Hello, my name is... (identify yourself), I am part of the team at the health unit where you participate in the Group... (name of the group). We missed you! Think about the importance of planning to achieve your goals, including weight loss. Until the next meeting (date, place and time)!*
